# Microneedle-mediated intelligent combination delivery for hair regeneration: A comprehensive review of technologies, mechanistic evidence, and translational prospects

**DOI:** 10.1016/j.mtbio.2026.103627

**Published:** 2026-08-31

**Authors:** Liang Lan, Yiyu Wang, Bin Xue, Peng Chen

**Affiliations:** Burn/Esthetic Plastic Surgery, The First Affiliated Hospital of Chongqing Medical University, No.1 Youyi Road, Yuzhong District, Chongqing, 400016, China

**Keywords:** Microneedle, Hair regeneration, Androgenetic alopecia, Transdermal delivery, Synergistic therapy, Intelligent delivery system, Closed-loop system

## Abstract

Androgenetic alopecia (AGA) is a prevalent hair-loss disorder with substantial psychosocial burden, while current treatments such as topical minoxidil and oral finasteride remain limited by insufficient follicular delivery, long-term adherence requirements, local irritation, systemic adverse effects, and recurrence after discontinuation. Microneedle (MN) technology offers a minimally invasive local intervention strategy by bypassing the stratum corneum, enhancing follicular exposure, reducing unnecessary systemic distribution, and providing mechanical stimulation to the scalp microenvironment. This review summarizes recent advances in MN-mediated intelligent combination delivery for hair regeneration, covering MN types, material selection, structural design, controlled-release strategies, and major therapeutic mechanisms, including antioxidative remodeling, pro-angiogenic support, growth factor and collagen delivery, immunomodulation, extracellular vesicle-based therapy, nanomedicine integration, and anti-androgenic intervention. We further discuss stimulus-responsive MN systems, including ROS-, pH-, enzyme-, temperature-, light-, ultrasound-, magnetic-, and electrically responsive platforms, as well as emerging closed-loop concepts that integrate sensing, data processing, and adaptive actuation. Importantly, we critically examine the gap between preclinical proof-of-concept studies and clinical translation, highlighting key challenges related to scalp-specific insertion, hair obstruction, payload stability, immune compatibility, scalable manufacturing, sterilization, patient usability, long-term safety, and regulatory uncertainty. By integrating mechanistic evidence with translational considerations, this review provides a structured perspective for researchers developing disease-tailored, microenvironment-oriented, and clinically feasible MN-based strategies for hair regeneration.

## Graphical abstract

**Figure:**
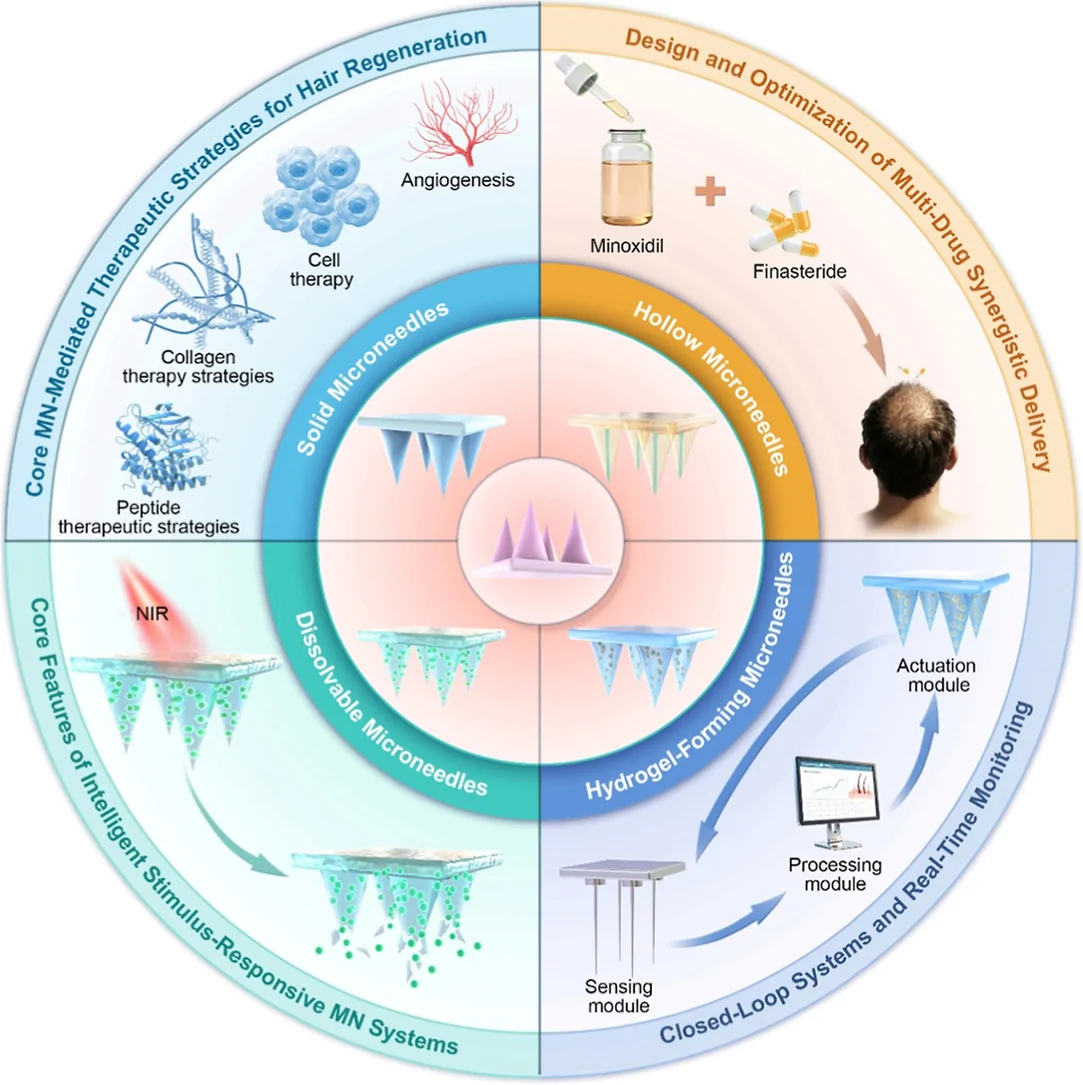

## 1 Introduction

Androgenetic alopecia (AGA) is the most prevalent subtype of alopecia, affecting approximately 15–30% of women and up to 50% of men worldwide [[1], [2], [3], [4]]. Beyond the loss of hair itself, AGA imposes a substantial psychosocial burden, frequently leading to reduced self-esteem, impaired social functioning, and mental health concerns, particularly among adolescents [5,6]. In recent years, clinical observations have suggested a trend toward earlier onset of alopecia, which may be associated with environmental pollution, lifestyle changes, and increasing psychosocial stress [7,8]. More broadly, alopecia comprises a heterogeneous group of hair disorders that, depending on the disease subtype, may arise from disruption of the hair follicle growth cycle, follicular miniaturization, immune dysregulation, or irreversible destruction of hair follicles [9].

The canonical pathogenesis of AGA is closely linked to androgen sensitivity within the follicular unit. Type II 5α-reductase converts testosterone into dihydrotestosterone (DHT), which binds to androgen receptors in dermal papilla cells and thereby promotes shortening of the anagen phase, prolongation of the telogen phase, and progressive follicular miniaturization [[9], [10], [11]]. However, AGA is increasingly recognized as a multifactorial disorder in which perifollicular oxidative stress, chronic low-grade inflammation, impaired angiogenesis, mitochondrial dysfunction, dermal papilla cell senescence, and insufficient activation of hair follicle stem cells collectively disturb the regenerative follicular niche [9,12]. Therefore, effective hair regeneration therapy should not only suppress androgen signaling or improve local blood perfusion, but also coordinate multi-pathway modulation of the pathological perifollicular microenvironment [12].

FDA-approved treatments primarily include topical minoxidil and oral finasteride, with oral baricitinib recently added [5]. However, these modalities have notable limitations. Minoxidil requires daily use, has low bioavailability, and can cause local irritation; topical minoxidil can also induce pruritus, erythema, scaling, dryness, irritant or allergic contact dermatitis, and unintended facial hypertrichosis, particularly when formulations contain irritating excipients such as propylene glycol [10,11,13]. Transient shedding during the early treatment phase may further reduce patient confidence and compromise adherence [10,11]. Finasteride may induce systemic adverse effects, including sexual dysfunction [14]; oral finasteride and dutasteride have also been associated with decreased libido, erectile or ejaculatory dysfunction, breast tenderness or gynecomastia, mood-related symptoms, and teratogenic risks in women of childbearing potential [10,11,15,16]. Although low-dose oral minoxidil has been increasingly used off-label for alopecia in recent years, it may cause dose-dependent hypertrichosis, ankle edema, tachycardia, headache, dizziness, and, in rare cases, more serious cardiovascular events [15,17]. The long-term safety of baricitinib warrants further evaluation [18,19]; for systemic Janus kinase inhibitors, clinically relevant safety concerns include acne, upper respiratory tract infections, herpes zoster, laboratory abnormalities, elevated lipid levels, and potential risks highlighted by warnings for serious infections, thromboembolic events, and malignancies [[18], [19], [20]]. Other therapeutic approaches, such as platelet-rich plasma, low-level laser therapy, and hair transplantation, are also constrained by high costs, protocol heterogeneity, insufficient standardization, invasiveness, or unstable long-term efficacy [9].

A major barrier to topical therapy is the stratum corneum, which limits drug penetration into deep hair follicles [7,21]. This barrier is particularly important for hydrophilic compounds, biologics, extracellular vesicles, natural bioactive ingredients, and nanomedicines, as these payloads often exhibit insufficient passive permeation and limited follicular retention after conventional topical administration [9,22,23]. By contrast, oral administration results in systemic exposure, thereby increasing the risk of off-target effects [24]. Therefore, the development of efficient, safe, and patient-friendly delivery systems represents an important direction in alopecia therapy [25]. Microneedles typically consist of arrays of micron-scale needles with lengths ranging from several hundred micrometers to approximately 1 mm, enabling disruption of the stratum corneum and formation of reversible microchannels for direct delivery into the epidermis, dermis, or perifollicular regions [9,22].

As next-generation transdermal delivery platforms, microneedles can penetrate the stratum corneum in a nearly painless manner, create microchannels, and directly deliver active agents into the dermis [7,21,22,[26], [27], [28]]. This strategy can enhance local bioavailability, reduce systemic exposure, provide mechanical stimulation, and enable intelligent delivery through stimulus-responsive designs [9,22,25,29,30]. In the context of hair regeneration, MN-induced mechanical microinjury may also trigger wound-healing cascades, promote angiogenesis and extracellular matrix remodeling, stimulate local growth factor release, and activate the Wnt/β-catenin signaling pathway, thereby facilitating the transition of hair follicles from telogen to anagen [9,31]. Depending on the materials and release mechanisms, MN systems can be designed as solid, coated, hollow, dissolvable, hydrogel-forming, core–shell, or multilayer platforms, thereby enabling flexible control of drug loading, release kinetics, tissue retention, and spatiotemporal distribution [9,22].

In recent years, MN technology has been evolving from a general-purpose transdermal delivery tool into a disease-tailored therapeutic platform. Disease-customized MN design emphasizes matching needle geometry, material composition, payload stability, pharmacokinetic requirements, pathological barrier characteristics, and targeting precision to specific disease needs [32]. This concept is highly relevant to alopecia treatment because the hair follicle niche is deeply located, dynamically regulated, and influenced by multiple pathological factors, including DHT accumulation, oxidative stress, inflammation, insufficient vascular supply, and stem cell dysfunction [9,12,32]. Accordingly, intelligent MN systems responsive to endogenous stimuli such as reactive oxygen species, pH, enzymes, or inflammatory mediators, as well as to exogenous stimuli such as near-infrared light, ultrasound, magnetic fields, temperature, or electrical stimulation, offer opportunities for controlled, on-demand release within the scalp microenvironment [22,32].

The diagnostic and monitoring capabilities of MNs further expand their potential in individualized alopecia management. MN-based sensing platforms can access dermal interstitial fluid and detect biochemical or physiological signals, including pH, ions, cytokines, oxidative stress markers, and drug concentrations [22,33]. When integrated with data-processing and actuation modules, such platforms may support closed-loop theranostic systems, enabling treatment intensity to be adjusted according to real-time changes in the follicular microenvironment [22]. Although these systems remain in the early stages of alopecia therapy, they provide a rational direction for future intelligent and personalized hair regeneration strategies [22,32].

Combination or potentially synergistic MN-mediated delivery has emerged as a promising strategy to address the multifactorial pathogenesis of AGA. Recent MN systems have combined vasodilators or anti-androgenic agents with nanoparticles, extracellular vesicles, antioxidants, pro-angiogenic factors, or natural bioactive compounds to simultaneously stimulate hair growth and remodel the perifollicular microenvironment [9,12,23]. However, in many studies, these effects are better interpreted as multi-pathway or additive benefits unless rigorous quantitative synergy analysis has been performed. For example, a rapidly dissolving MN patch capable of co-delivering minoxidil nanoparticles and *Platycladus orientalis* leaf-derived extracellular vesicles was reported to improve follicular targeting, reduce oxidative stress and inflammation, enhance angiogenesis, activate hair follicle stem cells, and promote telogen-to-anagen transition in AGA models [15]. Although this design supports multi-mechanistic intervention, whether the combined outcome represents true pharmacological synergy rather than additive or complementary effects requires further validation through appropriate single-component comparisons and quantitative synergy assessment. Compounds and vesicles derived from traditional medicine, such as flavonoids, saponins, polysaccharides, and plant-derived extracellular vesicles, have also expanded the therapeutic repertoire of MN-based hair regeneration systems owing to their multicomponent and multitarget bioactivities [23]. However, their poor permeability, insufficient stability, and difficulties in dose control further underscore the need for rational MN engineering [23].

In this review, we systematically summarize recent advances in MN-mediated intelligent combination delivery systems and potentially synergistic strategies for hair regeneration. We first introduce the pathological basis of alopecia and the design principles of MN platforms, including material selection, structural configuration, and release mechanisms. We then discuss major therapeutic strategies, including antioxidative modulation, pro-angiogenic therapy, regulation of growth factor and collagen signaling, cell- and extracellular vesicle-based therapy, and multi-drug combination delivery, including minoxidil–finasteride-based regimens and other systems with additive or potentially synergistic effects. Particular emphasis is placed on stimulus-responsive MNs, closed-loop sensing and actuation systems, and translational challenges related to mechanical integrity, payload stability, scalable manufacturing, safety evaluation, regulatory pathways, and patient adherence. By integrating disease-tailored engineering design with multi-target biological intervention, MN-based intelligent delivery systems may contribute to safer, more precise, and more personalized alopecia therapy, provided that their efficacy, safety, manufacturability, and clinical value are rigorously validated.

## 2 Core microneedle types and design

### 2.1 Solid microneedles

Solid microneedles are among the earliest MN designs, typically fabricated from metals, silicon, or rigid polymers to provide sufficient stiffness for reliable stratum corneum puncture and repeated mechanical stimulation [33,34]. They first create microchannels in the skin, followed by topical drug application, thereby allowing drugs to penetrate more deeply through these conduits [29,35]. Because solid MNs generally do not carry therapeutic agents within the needle body, their delivery performance primarily depends on the number, depth, and lifetime of the microchannels generated, as well as the intrinsic diffusion properties of the subsequently applied topical formulation [[36], [37], [38]]. Accordingly, the design of solid MNs should prioritize reliable insertion, which is governed by geometric parameters such as needle length, tip sharpness, base diameter, aspect ratio, needle shape, inter-needle spacing, and array density [39]. Sufficiently sharp tips can reduce the force required to puncture the skin. In contrast, overly slender needles or excessively dense arrays may compromise insertion efficiency due to needle bending, buckling, or nonuniform force distribution across the array [[40], [41], [42]].

In alopecia treatment, solid MNs are frequently used as “microneedle rollers,” which enhance drug permeation via physical puncture while providing mechanical stimulation, a factor supported by clinical and experimental evidence as a contributor to hair-growth improvement [43,44]. This dual action is highly relevant to hair regeneration because transient microinjury not only improves access of topical agents to perifollicular regions but may also activate wound-healing-related signaling, increase local blood flow, and promote perifollicular growth factor release. However, the regenerative benefit of mechanical stimulation is likely dependent on treatment intensity and frequency; excessive or poorly controlled rolling may instead aggravate irritation, bleeding, inflammation, or barrier disruption. For example, clinical and patient-oriented evidence from Zhang et al. indicated that combining microneedle rollers with topical therapy was associated with improved therapeutic outcomes and patient satisfaction in AGA [6,8]. From a delivery perspective, solid MNs are more suitable as pretreatment tools to enhance the follicular penetration of topical minoxidil, natural bioactive ingredients, anti-inflammatory agents, or nanoformulations, rather than as independent long-acting drug reservoirs [33,34,45,46].

The principal limitations of solid MNs include their two-step workflow—puncture followed by topical administration—and limited dose-control capability, which constrain their clinical utility [29,[36], [37], [38]]. Unlike hollow, dissolvable, or hydrogel-forming MNs, solid MNs exert only indirect control over drug release, because the actual delivered dose is influenced by micropore closure, formulation residence time, post-treatment occlusion conditions, and interpatient differences in administration behavior [33,36,38,47]. In scalp applications, residual hair shafts, sebum, curved skin surfaces, and regional differences in skin thickness may further reduce MN–skin contact and insertion uniformity. Therefore, optimizing roller needle length, rolling pressure, treatment frequency, and the combination with appropriate topical formulations is essential to improve reproducibility and reduce the risk of irritation, bleeding, or infection [44,48]. Overall, solid MNs have advantages in mechanical stimulation and pretreatment-enhanced permeation, but remain clearly limited in precise multi-drug loading, sustained release, and programmed sequential delivery [34,45,49,50].

### 2.2 Hollow microneedles

Hollow microneedles resemble miniature hypodermic needles with internal channels that directly deliver liquid formulations to targeted dermal depths [33,35,37]. Accordingly, their design must maintain lumen patency during both insertion and infusion, requiring careful optimization of needle length, tip geometry, lumen diameter, wall thickness, and mechanical stiffness to balance skin penetration, fluid transport, and structural integrity [34,48,51]. Unlike solid MNs, hollow MNs allow drug delivery to be regulated by infusion volume, flow rate, injection pressure, and administration duration, making them particularly suitable for controlled-dose delivery of soluble small molecules, biologics, growth factors, or vesicle-based formulations [33,47,52]. Wu et al. developed an ultrasound-assisted hollow MN array (USHM), in which acoustic cavitation markedly enhanced the transdermal delivery of finasteride and accelerated hair-cycle progression compared with controls in a preclinical experimental setting. This reported system illustrates that hollow MNs can be integrated with external physical stimulation to improve transcutaneous drug transport and potentially increase drug deposition in perifollicular regions [29,53].

Their advantages include precise control over dose and delivery rate, as well as favorable compatibility with liquid drugs and biologics [29]. For hair regeneration therapy, these features are particularly valuable when therapeutic payloads require quantitative administration or are difficult to encapsulate within dissolvable matrices. In principle, when connected to reservoirs, valves, pumps, or pressure-driven systems, hollow MNs can also support repeated, pulsatile, or continuous infusion [54,55]. However, key challenges include clogging, fluidic constraints, and fabrication complexity [35], as well as dependence on external pumps or pressure sources that increase operational complexity [30]. In addition, tissue compression at the needle orifice, formulation leakage or backflow, variation in insertion depth, and unstable contact with curved or hair-bearing scalp surfaces may all compromise dose reproducibility [48]. Therefore, anti-clogging lumen designs, stable applicator-assisted insertion, appropriate infusion pressure, and scalp-adapted patch fixation should be prioritized to advance hollow MNs toward practical alopecia therapy [56].

### 2.3 Dissolvable microneedles

Dissolvable microneedles are fabricated from biodegradable polymers such as hyaluronic acid, polyvinylpyrrolidone, and starch, with therapeutic agents encapsulated within the needle tips [29,57]. Their formulation design must balance mechanical strength before insertion with dissolution behavior after insertion, because polymer type, molecular weight, concentration, crystallinity, residual moisture, and potential crosslinking or blending strategies can substantially influence needle stiffness, insertion reliability, and matrix hydration [58,59]. Key geometric parameters, including needle height, tip sharpness, base diameter, array density, and backing flexibility, also determine whether the patch can puncture hair-bearing scalp with sufficient stability and maintain intimate skin contact [58,60].

After insertion, the MNs dissolve in interstitial fluid and release their payloads [61]. Because therapeutic cargoes are embedded within a soluble matrix, drug release is jointly governed by interstitial fluid uptake, polymer swelling or erosion, drug diffusion, and the spatial distribution of the payload within the needle tips or needle body [49,52,62]. Accordingly, dissolvable MNs can be engineered as rapid-release, sustained-release, or staged-release systems by tuning polymer composition, drug-loading position, needle architecture, and backing design [50,63]. Zhou et al. developed a flexible-backed, dissolvable MN patch loaded with cedrol, enabling sustained dermal delivery and increasing perifollicular drug concentrations [64]. This design is highly relevant to alopecia therapy because a flexible backing improves patch conformity to the curved scalp surface. In contrast, localized tip loading reduces drug loss from the skin surface and enhances deposition near the follicular niche [65]. Nevertheless, improved conformity does not necessarily guarantee uniform insertion across the hair-bearing scalp, where hair shafts, sebum, and regional curvature may still impair patch–skin contact.

Dissolvable MNs offer several advantages, including one-step application, absence of biohazardous sharps waste, no need for retrieval, and tunable release profiles [7]. Compared with solid MN pretreatment, this one-step format integrates the drug reservoir and puncturing structure into a single soluble needle array, thereby improving usability and dose localization [66]. Available preclinical and emerging clinical evidence suggests that dissolvable MNs may improve alopecia-related therapeutic outcomes and reduce the frequency of administration compared with conventional topical administration [18,67]. Nevertheless, their performance remains constrained by limited drug-loading volume within the needle body, possible incomplete insertion or dissolution, humidity-sensitive storage stability, and the need to maintain uniform drug distribution between the needle tips and backing layer [68]. Therefore, rational optimization of polymer mechanical properties, dissolution kinetics, loading efficiency, and scalp-adapted patch adhesion is essential to achieve reproducible hair regeneration therapy [59,69].

### 2.4 Hydrogel-forming microneedles

Hydrogel-forming microneedles consist of crosslinked polymer networks with dual functionality for drug delivery and interstitial fluid extraction in biosensing [70]. Unlike dissolvable MNs, they generally do not fully degrade after insertion; instead, they absorb interstitial fluid, forming swollen hydrogel conduits that connect the superficial skin layers to an external drug reservoir or sensing layer [66,71]. Their design therefore requires simultaneous consideration of polymer composition, crosslinking density, swelling ratio, mesh size, needle height, tip sharpness, base diameter, and backing flexibility, as these parameters collectively determine dry-state puncture strength, in-skin swelling behavior, fluid uptake capacity, and molecular diffusion efficiency [58,59].

After insertion, the MNs absorb tissue fluid and swell, enabling long-acting drug release through matrix swelling, degradation, or diffusion pathways, which is well suited to the need for prolonged dosing in hair regeneration [28,70,72,73]. However, prolonged release is not inherently advantageous unless the release duration, local concentration, and residence time match the biological window of follicular response. The release profile can be further optimized by regulating hydrogel network density, degradable chemical linkages, drug loading in the reservoir layer, nanoparticle incorporation, and drug diffusion coefficients, thereby enabling long-term or sequential delivery at follicular sites [59,68]. Ding et al. engineered hydrogel-forming MNs that co-deliver vascular endothelial growth factor (VEGF) and ritlecitinib/polyhydroxyalkanoate nanoparticles for androgenetic alopecia intervention, demonstrating enhanced skin-insertion capability and minimally invasive, long-acting delivery in a preclinical study [19]. This co-delivery strategy is highly aligned with hair regeneration requirements: VEGF helps sustain a pro-angiogenic perifollicular microenvironment, ritlecitinib-loaded nanoparticles provide prolonged immune modulation, and the nanoparticle-composited hydrogel network functions as a reservoir to maintain stable local drug concentrations around hair follicles [74]. However, this type of multi-component hydrogel MN system should be interpreted primarily as a combination delivery unless quantitative evidence demonstrates that the joint effect exceeds the expected additive response.

**Fig. 1:**
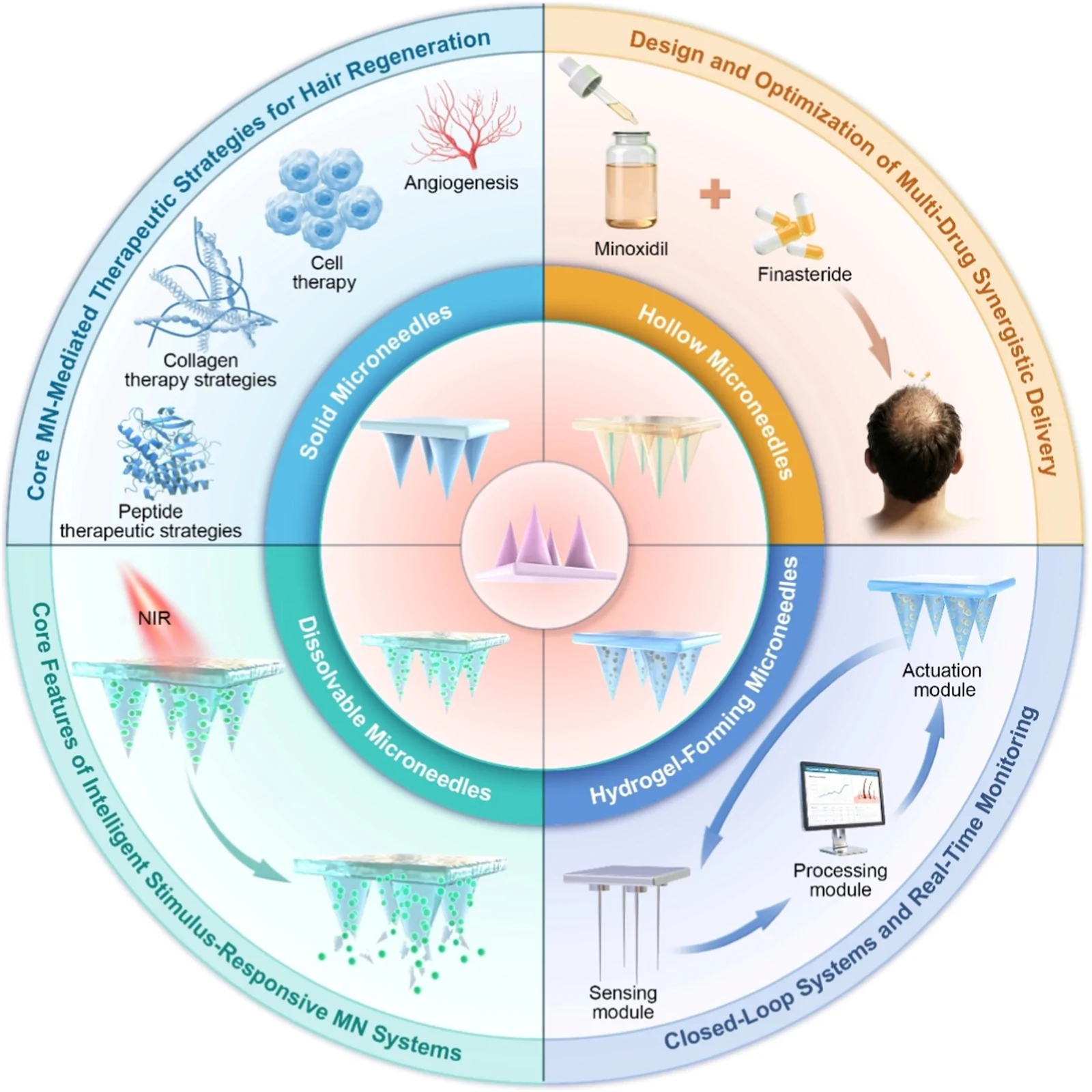
**A review framework of microneedle-mediated intelligent synergistic delivery systems for hair regeneration.** This schematic systematically outlines the core content and logical structure of this review, encompassing: (1) the fundamental types and designs of microneedle technologies; (2) core therapeutic strategies based on different mechanisms (e.g., antioxidation, pro-angiogenesis, growth factors); (3) the design and optimization of multi-drug synergistic delivery systems to enhance efficacy; (4) the features of intelligent stimulus-responsive microneedle systems for precise drug release; and (5) the future development of closed-loop theranostic systems integrating real-time monitoring and feedback control.

Although hydrogel-forming MNs hold considerable promise for integrated theranostics, their fabrication process is complex, and production costs remain relatively high [70]. Additional translational challenges include uniformity of swelling behavior, batch-to-batch stability of drug loading, premature drug leakage, excessive interstitial fluid influx, adhesion performance on curved and hair-bearing scalp, and comfort during long-term application [56,75] (see Fig. 1).

**Fig. 2:**
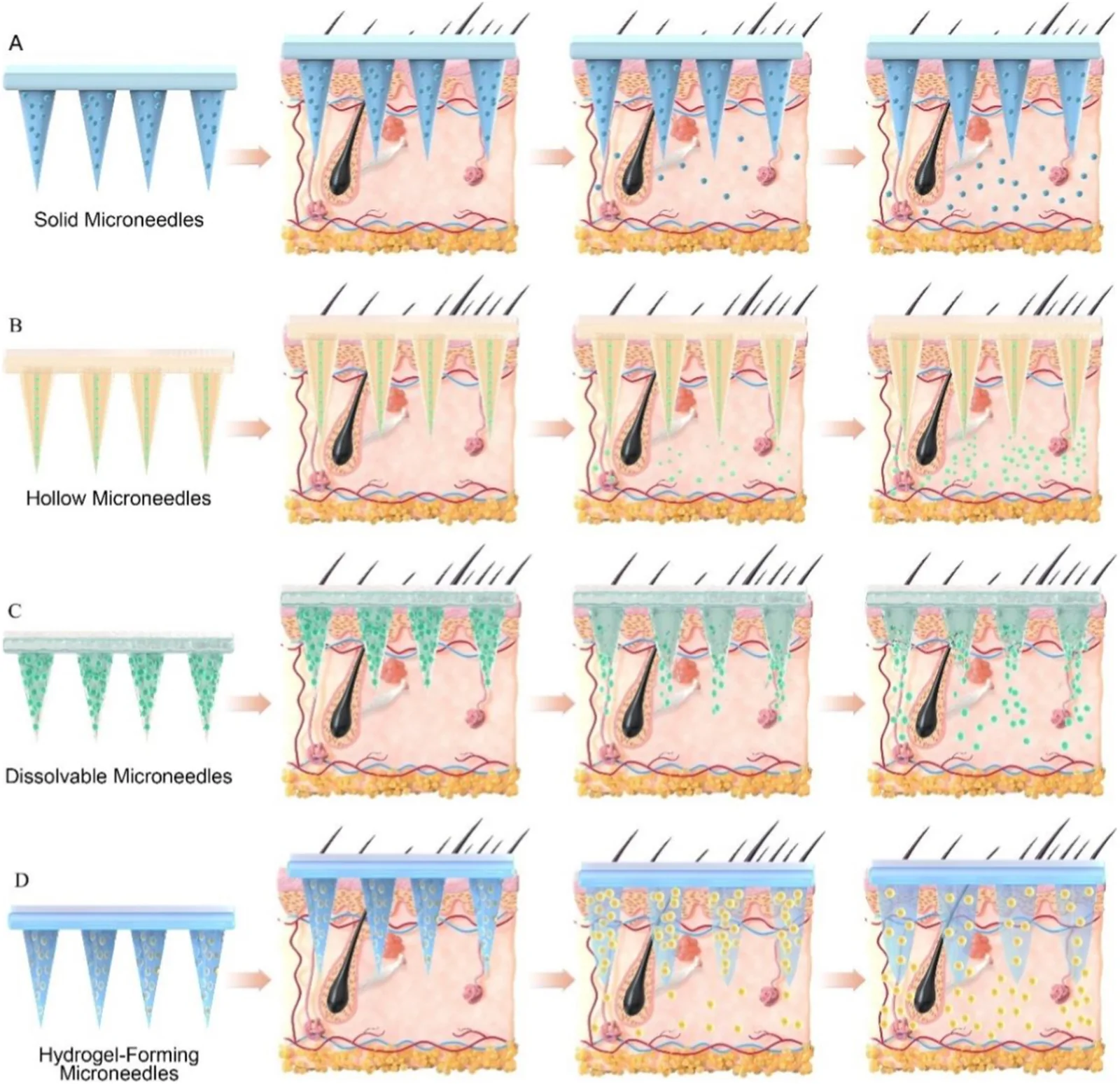
**Schematic illustration of four core types of microneedle delivery systems and their working mechanisms.** (A) Solid microneedles: pre-puncture the skin to create microchannels for subsequent drug permeation. (B) Hollow microneedles: feature an internal cavity for precise injection of liquid formulations. (C) Dissolvable microneedles: fabricated from biodegradable materials that dissolve within the skin to release the encapsulated payload. (D) Hydrogel-forming microneedles: swell upon uptake of interstitial fluid, forming a hydrogel matrix that enables sustained drug release or biomarker extraction.

In summary, solid, hollow, dissolvable, and hydrogel-forming microneedles each possess distinct characteristics in materials, design principles, and drug-release mechanisms (Table 1 and Fig. 2), offering diverse and precise delivery options for hair regeneration therapy. Among them, hydrogel-forming microneedles are particularly suited for long-acting and theranostic applications; solid microneedles are more appropriate for pretreatment-enhanced permeation, hollow microneedles for controlled liquid infusion, and dissolvable microneedles for convenient one-step local drug deposition.

**Table 1:** Comparison of the four core types of microneedles.

Type	Administration Approach	Delivery Efficiency	Advantages	Disadvantages	Ref.
Solid MNs	Poke-patch (two-step)	Drug loading: + Sustained release: +	• High mechanical strength and physical stability • Well-established fabrication • Mechanical stimulation promotes hair growth	• Two-step operation, inconvenient • Poor dose accuracy • Reuse may pose an infection risk	[29,35,36]
Hollow MNs	Poke-flow (continuous injection)	Drug loading: ++++ Sustained release: ++++	• Precise control over dose and infusion rate • Compatible with liquid drugs and biologics • Can integrate with external enhancement (e.g., ultrasound)	• Susceptible to clogging and fluidic constraints • Complex fabrication • Requires external pump/pressure source	[30,35]
Dissolvable MNs	Poke-release (dissolution)	Drug loading: ++++ Sustained release: ++++	• One-step application, user-friendly • No biohazardous sharps waste, no retrieval • Tunable release profiles • High biocompatibility	• Relatively low mechanical strength • Stringent requirements for matrix materials • Release duration is relatively fixed	[7,33]
Hydrogel-Forming MNs	Poke-release (swelling)	Drug loading: ++++ Sustained release: ++++	• Dual functionality: drug delivery and ISF extraction • Enables long-acting sustained release • Potential for personalized therapy and monitoring	• Limited mechanical strength • Complex fabrication, higher cost • Risk of interstitial fluid ingress	[34,41,42]

#### 2.4.1 Material selection in AGA-oriented microneedle design

Commonly used polymers include hyaluronic acid (HA), polyvinylpyrrolidone (PVP), hyaluronic acid methacrylate (HAMA), chitosan, gelatin, and polyvinyl alcohol (PVA), which exhibit favorable biocompatibility, biodegradability, and sufficient mechanical strength [29,76]. For androgenetic alopecia (AGA)-oriented microneedles (MNs), material selection should be matched to the scalp microenvironment, follicular target depth, physicochemical properties of therapeutic payloads, and the requirement for long-term repeated administration. Unlike acute wound treatment or vaccination, AGA therapy requires sustained or repeated modulation of perifollicular angiogenesis, inflammation, oxidative stress, androgen signaling, and hair follicle stem cell activity. As a third-generation transdermal drug delivery platform, MNs can disrupt the stratum corneum barrier at the microscale and deliver macromolecules, biologics, and nanocarriers that are difficult to penetrate using conventional topical formulations [7,77]. Therefore, MN materials should not be evaluated solely by puncture capability but should also be comprehensively assessed for follicular deposition, payload stability, local retention, release duration, scalp tolerability, and manufacturability. Representative materials, applicable MN types, key design features, AGA application potential, and major limitations are summarized in Table 2.

**Table 2:** Summary of material characteristics for AGA-oriented microneedles.

MN type	Representative materials	Key material characteristics	AGA-oriented design rationale	Suitable cargoes or uses	Main limitations of the AGA application	Ref.
**Solid MNs**	Stainless steel, titanium, silicon, ceramics, rigid resins, PMMA, PLA	High modulus, strong puncture ability, dimensional stability, fracture resistance, and limited intrinsic drug-loading capacity	Suitable for reproducible scalp microporation and mechanical stimulation; may enhance subsequent penetration of topical or nanocarrier formulations and activate wound-healing-related signals involved in hair cycling	Pretreatment before topical minoxidil/finasteride, mechanical stimulation, and permeability enhancement for anti-inflammatory agents or nanoformulations	Two-step administration; limited drug loading and release control; dose affected by microchannel closure, formulation spreading, hair shafts, sebum, and scalp contact; possible irritation or fracture risk	[33,34,44]
**Hollow MNs**	Stainless steel, silicon, rigid resins, high-strength polymers, ceramics	Lumen-containing structure, high wall stiffness, tip sharpness, infusion compatibility, pressure-driven delivery	Suitable when controlled intradermal infusion is needed; can reduce surface drug loss and deliver liquid or higher-dose formulations closer to follicular regions	Liquid formulations; growth factors; JAK inhibitors; extracellular vesicles; nanoparticle suspensions; relatively high-dose agents	Complex fabrication; lumen blockage by viscous formulations, particles, vesicle aggregation, or compressed scalp tissue; risk of leakage, backflow, and uneven infusion	[34,47,51]
**Dissolvable MNs**	HA, PVP, gelatin, chitosan, sodium alginate, sodium hyaluronate, starch-based polymers, PVA blends	Hydrophilic, biodegradable, drug-loaded needle matrix, hydration-triggered dissolution, tip-localized loading, tunable dissolution by polymer composition	Suitable for one-step follicular deposition; reduces drug loss on scalp surface and improves localization around follicular units; useful for repeated outpatient or self-administered AGA therapy	Minoxidil; finasteride; JAK inhibitors; phytochemicals; peptides; nucleic acids; liposomes; polymeric nanoparticles; LPNs	Lower dry-state strength than rigid MNs; humidity-sensitive storage; release affected by hydration and local interstitial fluid; overly fast dissolution may cause superficial release, whereas slow dissolution may delay perifollicular exposure	[33,44,78,79]
**Hydrogel MNs**	HA/HAMA, PVA, PEG/PEGDA, GelMA, alginate, collagen, silk fibroin, PMVE/MA	Crosslinked polymer network, dry-state insertion strength, post-insertion swelling, hydrated diffusion pathways, tunable mesh size, and release behavior	Suitable for sustained local exposure and long-acting AGA therapy; can prolong retention near follicular niches and potentially reduce dosing frequency	VEGF; growth factors; JAK inhibitors; immunomodulators; exosomes; nanoparticle formulations; sustained delivery systems	Requires a balance between crosslinking, strength, swelling, and diffusion; excessive crosslinking may restrict release, insufficient crosslinking may cause weak insertion, leakage, or inconsistent residence time; more demanding quality control	[54,70,[79], [80], [81], [82]]

For solid MNs, material systems primarily serve to ensure reproducible mechanical interactions with the scalp rather than to function as intrinsic drug reservoirs. Metals, silicon, ceramics, resins, and rigid polymers possess high modulus, good dimensional stability, and sufficient fracture resistance, allowing them to form transient microchannels across the stratum corneum [33,34,45]. In AGA therapy, this feature can enhance the subsequent penetration of topical minoxidil, finasteride, anti-inflammatory agents, or nanocarrier formulations. It may also activate wound-repair-related signals that regulate the hair cycle through controlled mechanical stimulation. Solid MNs generally employ a two-step “puncture-then-apply” administration mode, and their therapeutic efficacy is highly dependent on the spreading behavior of subsequently applied formulations, microchannel closure kinetics, and contact uniformity on hair-bearing scalp [33,34]. Hair shafts, sebum, and curved scalp surfaces may further reduce dose consistency. Although rigid solid MNs exhibit excellent insertion performance, tip fracture, skin irritation, and dose deviation caused by rapid microchannel closure are unfavorable for long-term use in chronic alopecia [34,45]. Therefore, solid MN materials are more suitable for pretreatment or mechanical stimulation strategies; when prolonged follicular exposure is required, their limitations in drug-loading capacity and programmable release should be fully considered.

For hollow MNs, material selection should focus on maintaining lumen integrity during insertion and infusion. Nondegradable metals, silicon, rigid resins, and high-strength polymers can provide sufficient needle-wall stiffness and tip sharpness, allowing the needles to resist deformation during penetration into scalp tissue [34,47,51]. Hollow structures can be connected to external driving devices to achieve controlled infusion and are suitable for delivering high-dose drugs and macromolecular bioactive liquid formulations [47,51]. Therefore, they have potential value for AGA therapies requiring liquid formulations, growth factors, extracellular vesicles, or nanoparticle suspensions. Compared with passive topical delivery, hollow MNs may enable more controlled intradermal input and reduce drug loss on the scalp surface. Nevertheless, AGA-oriented hollow MNs also face formulation-related limitations: highly viscous solutions, aggregated nanoparticles, insufficient vesicle stability, or dermal tissue compression may clog the lumen or cause backflow and leakage [47,51]. Thus, needle material, lumen geometry, formulation viscosity, particle size distribution, and infusion pressure should be considered as an integrated system rather than evaluated in isolation.

For dissolvable MNs, hydrophilic biodegradable polymers simultaneously serve as the needle framework and drug reservoir [33,78]. HA, PVP, gelatin, chitosan, sodium alginate, sodium hyaluronate, starch-based polymers, and their blends can encapsulate therapeutic agents within the needle tips and slowly dissolve, releasing payloads after hydration with interstitial fluid [78,[83], [84], [85]]. This one-step delivery format is highly relevant to AGA because it allows drugs to be placed directly near follicular units. Still, its advantage over conventional topical administration should be interpreted primarily in terms of improved local deposition rather than as automatic evidence of superior clinical efficacy. Its broad loading capacity makes it suitable for small molecules such as minoxidil, finasteride, and JAK inhibitors, as well as phytochemicals, peptides, nucleic acids, and nanocarrier-based therapeutics [33,44]. However, the hydrophilicity that enables dissolution can reduce dry-state insertion strength and increase sensitivity to storage humidity [78,86]. Therefore, polymer molecular weight, concentration, crystallinity, residual moisture, glass-transition behavior, and blending ratio should be optimized to balance mechanical stability, localized tip loading, dissolution rate, and storage stability. Since the release behavior of dissolvable MNs is jointly determined by the material and the tissue microenvironment, controlled release remains challenging [78]. In AGA, an ideal dissolvable matrix is not simply one that dissolves “as rapidly as possible”; rather, it should release payloads at a rate favorable for perifollicular deposition while avoiding premature release near the scalp surface or delayed exposure around the follicular niche.

For hydrogel-forming MNs, crosslinked polymer networks provide a material platform for sustained delivery and prolonged perifollicular exposure [77,79]. Crosslinked hydrogels based on HA/HAMA, PVA, PEG/PEGDA, GelMA, alginate, collagen, and silk fibroin exhibit sufficient puncture stiffness in the dry state; upon insertion into the skin, they absorb interstitial fluid and swell, forming continuous hydrated diffusion pathways [79,80]. The concentration gradient generated by swelling can continuously drive drug diffusion toward target tissues, enabling long-acting local retention [79]. Their swelling and release behaviors can be regulated by crosslinking density, polymer concentration, molecular weight, charge properties, mesh size, degradation rate, and drug–polymer interactions [81,82]. These parameters are highly relevant to AGA because VEGF, JAK inhibitors, immunomodulators, exosomes, or nanoparticles may require repeated or sustained local exposure. Compared with rapidly dissolving MNs, hydrogel-forming MNs combine high drug loading, favorable biocompatibility, and long-acting controlled release, making them more suitable for the long-term management of chronic alopecia [54,77,79]. However, hydrogel materials require a finely balanced design: excessive crosslinking may improve insertion strength and dimensional stability but restrict swelling and molecular diffusion, whereas insufficient crosslinking may cause inadequate penetration, excessive swelling, drug leakage, or inconsistent residence time. Therefore, hydrogel-forming MN materials for AGA should be optimized according to the target residence time, drug diffusion profile, and scalp comfort during repeated application.

Across these four MN categories, the core material challenge is to match mechanical performance with AGA-specific therapeutic requirements. Solid and hollow MNs rely more on rigid materials to ensure penetration or infusion. In contrast, dissolvable and hydrogel-forming MNs rely on polymer chemistry to integrate puncture resistance, drug loading, degradation/swelling, and controlled release [54]. Natural polymers such as HA, chitosan, gelatin, alginate, collagen, and silk fibroin offer favorable biocompatibility, biodegradability, skin affinity, and extracellular matrix-like characteristics, thereby supporting repeated perifollicular application [[83], [84], [85]]. Synthetic polymers such as PVP, PVA, PEG derivatives, PEGDA, PLA, PLGA, and rigid resins provide greater tunability in mechanical strength, dissolution behavior, elasticity, degradation, and manufacturing reproducibility [87,88]. Therefore, natural–synthetic composite matrices can balance biocompatibility, needle mechanics, and controllable release, making them highly suitable for AGA MN development [70,89].

Material selection should also be integrated with fabrication and translational requirements. Micromolding is suitable for many dissolvable and hydrogel-forming MNs because it enables control over tip filling, array geometry, and drug distribution, whereas 3D printing and precision molding can support more personalized needle dimensions or rigid hollow structures [[90], [91], [92]]. However, each material system must be evaluated for processing viscosity, drying or crosslinking conditions, residual solvents or monomers, sterilization compatibility, dose uniformity, long-term storage stability, and mechanical integrity after packaging. For AGA, additional use-related factors should also be considered, including adherence to the curved scalp, interference from hair shafts and sebum, patient comfort, and the feasibility of repeated self-administration. Overall, material selection for AGA-oriented MNs should integrate insertion reliability, follicular localization, payload protection, controlled release, and scalable manufacturing [77], rather than relying solely on general material biocompatibility or puncture strength.

#### 2.4.2 Structural design strategies for programmed and follicle-oriented delivery

Beyond basic designs, advanced programmed architectures are driving microneedles (MNs) toward greater precision and multifunctionality [77]. For AGA, structural programming is particularly important because hair regeneration generally requires the coordinated regulation of multiple biological processes, including perifollicular angiogenesis, inflammatory or immune activity, androgen-related signaling, oxidative stress, and activation of hair follicle stem cells [7,77,93].

Core–shell and multilayer MNs can achieve temporally programmed release. A degradable shell can function as a gating structure, allowing encapsulated agents to be released in stages at different rates and time points, thereby optimizing the temporal coordination of multi-agent delivery rather than necessarily demonstrating true pharmacological synergy [81,94]. For example, a rapidly dissolving outer layer may provide early anti-inflammatory or barrier-modulating effects. In contrast, a slowly degrading core or backing reservoir can maintain the release of growth factors, JAK inhibitors, or follicle activators over a longer period [80]. Such spatial compartmentalization also helps reduce premature interactions between incompatible payloads and improves the stability of biologics or nucleic acid therapeutics before release.

Embedding nanocarriers, including liposomes, nanoparticles, and nanofibers, into MNs is an important strategy for precise and intelligent delivery [[95], [96], [97], [98], [99]]. Nanocarriers can protect payloads from degradation, increase local concentrations, and enable controlled release [95,97]. This strategy is highly relevant to AGA because many hair-regenerative payloads, such as miRNAs, phytochemicals, peptides, proteins, extracellular vesicles, and hydrophobic small molecules, suffer from poor passive skin permeation or insufficient stability in conventional topical formulations [77,93]. Nanocarrier-integrated MNs combine the penetration advantage of MNs with the stabilization and sustained-release capabilities of nanosystems, thereby increasing drug retention around follicular units and reducing drug loss on the scalp surface [74,80]. For example, lipid–polymer hybrid nanoparticles encapsulating miR-218 have been loaded into dissolving MNs to activate the Wnt/β-catenin pathway and promote hair regeneration in experimental hair-regeneration models [95]. Electrospun nanofibers, with high specific surface area and tunable three-dimensional morphology/porosity, show potential for drug delivery, tissue engineering, and wound repair [98,99]. When incorporated into MN platforms, nanofibers may further provide structural reinforcement, additional drug reservoirs, or extracellular matrix-like cues that support perifollicular tissue remodeling [100].

Modular MNs can integrate rapid- and sustained-release layers within a single system to meet complex therapeutic needs, such as rapid release of anti-inflammatory agents followed by slow release of growth factors to coordinate hair regeneration [94]. However, increasing structural complexity may reduce dose uniformity, complicate sterilization and storage, and make it more difficult to determine each module's contribution. In this module-driven strategy, each component can be assigned a distinct function, including immediate modulation of the scalp microenvironment, sustained stimulation of perifollicular regions, or delayed maintenance therapy. Release profiles can be programmed by adjusting polymer composition, crosslinking density, drug distribution between the needle tips and backing layer, nanoparticle degradation rate, and reservoir architecture [79,80]. For clinical translation in AGA, these structural designs should also ensure dose uniformity, mechanical integrity, adequate adhesion to curved and hair-bearing scalp, minimal residue or leakage, and compatibility with scalable manufacturing [90]. Overall, AGA-oriented MN design should integrate material biocompatibility, insertion mechanics, follicular targeting, and programmed release, rather than relying on a single material or geometric parameter alone [7,77]. For multi-agent systems, future studies should further determine whether improved outcomes reflect additive, complementary, or truly synergistic effects through appropriate single-agent controls and quantitative combination analysis.

## 3 Core MN-mediated therapeutic strategies for hair regeneration

### 3.1 Antioxidative strategies

#### 3.1.1 Role of ROS in alopecia

Excessive reactive oxygen species (ROS) are closely implicated in the pathophysiology of AGA [101,102]. Oxidative stress damages follicular stem cells, suppresses proliferation, accelerates follicular regression, and precipitates premature entry into telogen [103,104]. ROS also impair peri-follicular microvasculature, further deteriorating the microenvironment [43]. Yuan et al. showed that ROS accumulation in preclinical AGA models suppresses stem cell function, whereas ROS clearance improves the niche and promotes regrowth [101]. Targeting ROS—especially via MN-assisted localized delivery of antioxidants—has thus become a promising strategy. However, its therapeutic value depends on achieving appropriate redox modulation rather than indiscriminate ROS elimination [102].

#### 3.1.2 MN delivery of nanozymes and antioxidants

**Fig. 3:**
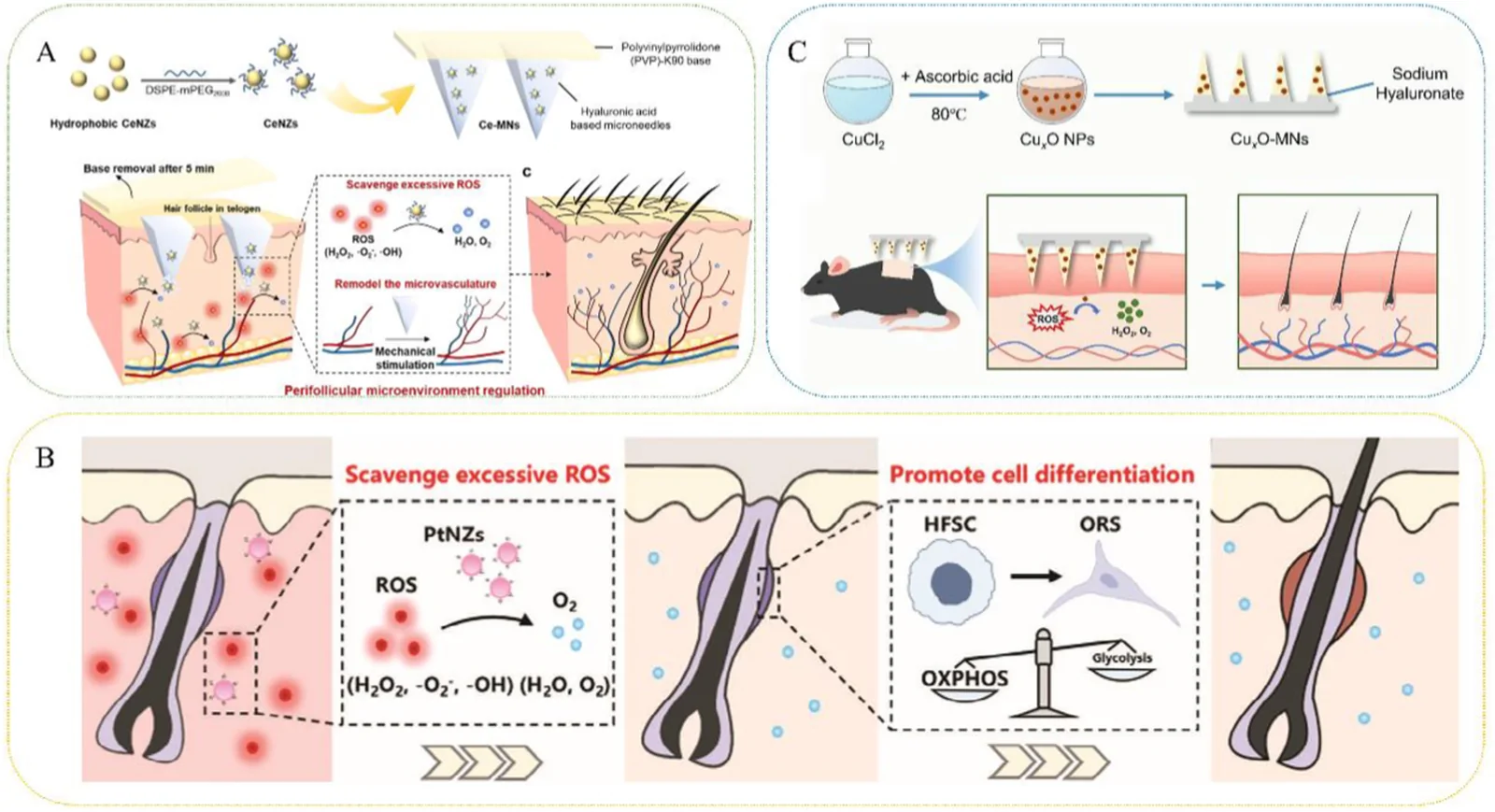
**Mechanism of nanozyme-loaded microneedle systems in promoting hair regeneration by regulating oxidative stress.** (A) Cerium nanozyme (CeNZs)-integrated microneedles scavenge excessive ROS around hair follicles and promote angiogenesis via mechanical stimulation, thereby improving the perifollicular microenvironment. Reproduced with permission from Ref. [101]. Copyright 2021 American Chemical Society. (B) Platinum nanozyme (PtNZs)-based microneedles catalytically reduce ROS to oxygen, which elevates local oxygen concentration, enhances oxidative phosphorylation in hair follicle stem cells, and promotes their differentiation into hair follicle lineage cells. Reproduced with permission from Ref. [103]. Copyright 2024, Wiley. (C) Copper oxide nanozyme (Cu_x_O)-loaded microneedles directly eliminate ROS, modulating oxidative stress levels around hair follicles to stimulate hair regrowth. Reproduced with permission from Ref. [102]. Copyright 2025, The Royal Society of Chemistry.

**Fig. 4:**
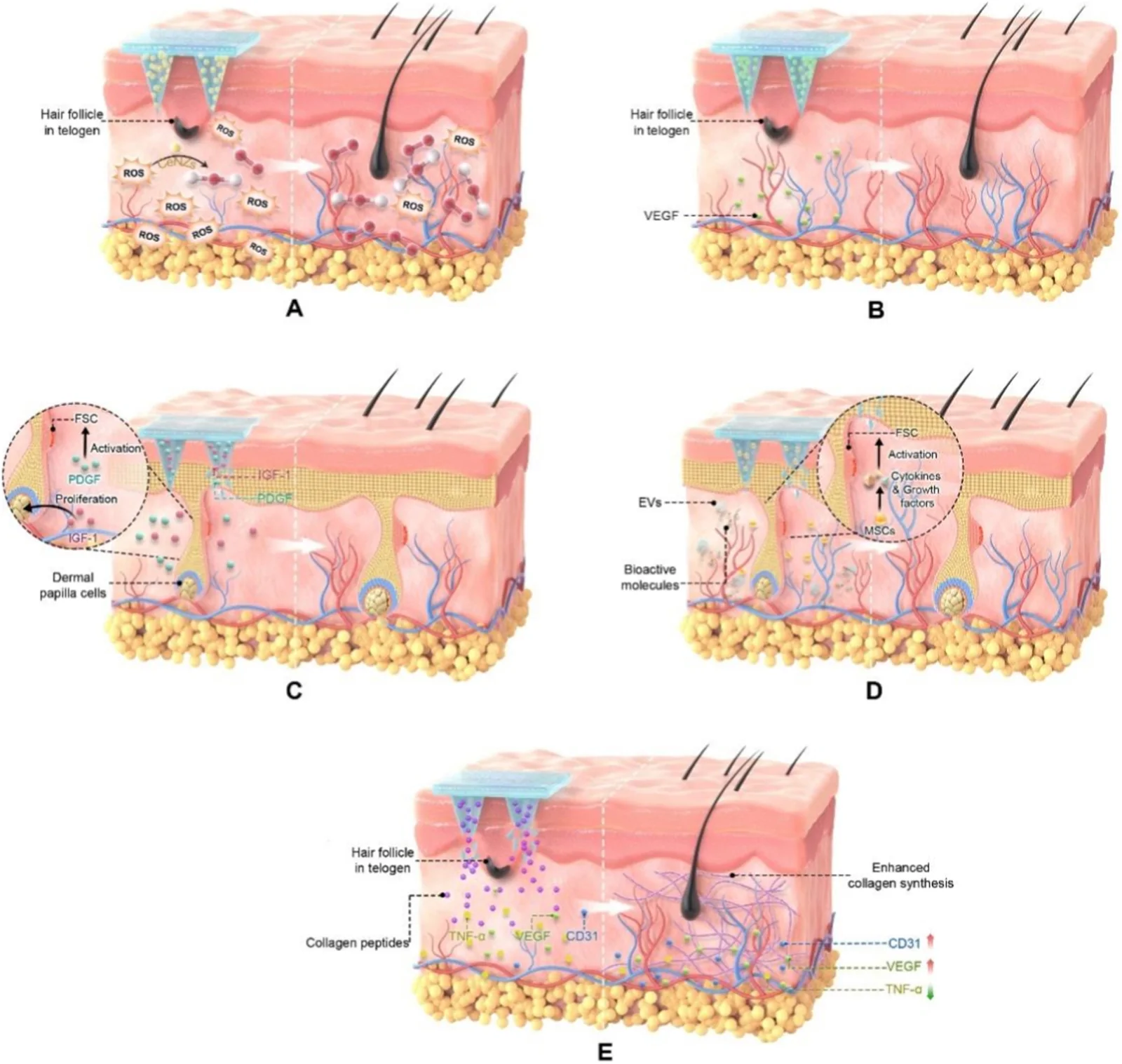
**Schematic illustration of the core therapeutic strategies for microneedle-mediated hair regeneration.** (A) **Antioxidant strategy**: Delivery of antioxidants such as nanozymes (e.g., CeNZs) to scavenge excess reactive oxygen species (ROS) around hair follicles, thereby ameliorating the oxidative stress microenvironment. (B) **Angiogenesis strategy**: Delivery of pro-angiogenic factors like vascular endothelial growth factor (VEGF) to promote perifollicular neovascularization, enhancing the supply of nutrients and oxygen. (C) **Growth factor strategy**: Delivery of key growth factors, including insulin-like growth factor-1 (IGF-1) and platelet-derived growth factor (PDGF), to directly activate hair follicle stem cells, stimulate dermal papilla cell proliferation, and regulate the hair growth cycle. (D) **Cell therapy strategy**: Delivery of cellular products such as stem cells and exosomes to systemically repair the follicular niche through multifaceted mechanisms, including paracrine signaling and immunomodulation.

Combining MNs with nanozyme antioxidants is an innovative approach [101,103]. Machine-learning-aided discovery identified a manganese thiophosphate (MnPS3) superoxide dismutase (SOD) mimic with an IC50 of 3.61 μg mL−1—12-fold lower than most reported SOD-like nanozymes [105]. An MnPS3 MN patch (MnMNP) was reported to scavenge local ROS and promote regrowth in experimental hair-regeneration models. Ceria nanozymes (CeNZ) exhibit catalase- and SOD-like activities; in AGA mouse models, CeNZ-loaded MNs (Ce-MNs) cleared peri-follicular ROS and, together with MN mechanical stimulation, promoted local VEGF expression and angiogenesis, thereby supporting combined modulation of oxidative stress and perifollicular vascular responses in AGA mice (Fig. 3A) [101]. Hu et al. designed platinum nanozyme-loaded dissolving MNs (Pt-MNs) that reduce ROS to O_2_ through a cascade-enzyme reaction, alleviating oxidative stress, and the resulting O_2_ further elevated oxidative phosphorylation (OXPHOS) in hair follicle stem cells (HFSCs), driving their differentiation toward outer root sheath (ORS) cells (Fig. 3B) [103]. Copper oxide nanozyme (Cu_x_O) MNs, synthesized via a green chemistry route, likewise demonstrated potent •OH and •O_2_^−^ scavenging ability, reducing oxidative stress while promoting vascular regeneration (Fig. 3C) [102]. In these preclinical studies, the systems provided more sustained local effects and a lower dosing frequency than standard minoxidil administration under the conditions tested (Fig. 4A) [[101], [102], [103]]. However, antioxidative MN strategies should be interpreted with caution. ROS are not solely detrimental molecules but also participate in physiological signaling during tissue repair and follicular cycling. Therefore, excessive or prolonged ROS depletion may not always be beneficial, and the long-term scalp retention, biodegradation, and immunological safety of nanozymes require further evaluation.

### 3.2 Angiogenesis strategies

#### 3.2.1 Importance of the peri-follicular microvasculature

Integrity and function of microvascular networks around follicles are essential, ensuring oxygen and nutrient supply [31,101]. Alopecic regions often show reduced vascular density and function [19]. Yuan et al. reported peri-follicular hypovascularity in AGA, a major contributor to niche deterioration [31]. Vasculature not only supplies nutrients but modulates hair cycle transitions, promoting the shift from telogen to anagen [31,43]. MNs enable precise delivery of pro-angiogenic agents [19,31].

#### 3.2.2 MN delivery of pro-angiogenic factors

Dissolving MNs delivering bimodal vasodilator M119 stimulated local circulation and promoted regrowth [3]. Yuan et al. designed dissolving MNs integrating MSC-conditioned medium (CM-MNs), enriched with angiogenic factors regulated by HIF-1α, which enhanced neovascularization, stem-cell activation, and regrowth in preclinical AGA models [31]. Ding et al. developed hydrogel MNs carrying VEGF to promote peri-follicular angiogenesis and achieved superior hair-regeneration outcomes compared with minoxidil under the tested AGA model conditions [19]. Platelet-derived growth factor (PDGF)-rich MNs similarly enhanced angiogenesis [106]. MN-mediated pro-angiogenic delivery may offer a minimally invasive and more acceptable alternative to injections. Still, its practical advantage depends on dose control, factor stability, and avoidance of excessive or off-target vascular stimulation (Fig. 4B) [19,31,106]. It should also be noted that AGA is not simply a hypovascular disorder. Increased vascular markers or neovascularization in animal models may support follicular regeneration, but these endpoints do not necessarily predict durable improvements in human hair density. In addition, the dose window, stability, and potential off-target vascular effects of pro-angiogenic factors remain important translational concerns.

### 3.3 Growth factor strategies

#### 3.3.1 Roles of key growth factors in hair growth

Growth factors orchestrate follicle development and cycle regulation [106,107]. PDGF, VEGF, transforming growth factor-β (TGF-β), and insulin-like growth factor-1 (IGF-1) have been implicated [106,108]. IGF-1 promotes dermal papilla cell proliferation/migration; VEGF mainly drives peri-follicular vascularization; PDGF activates follicular stem cells and telogen-to-anagen transition [106,108]. Instability and poor skin permeation of protein therapeutics limit conventional delivery [107].

#### 3.3.2 MN delivery of growth factors

A borate-modified recombinant collagen XVII (COL17) MN system loading IGF-1 was developed for AGA. Given COL17's role in follicular aging and IGF-1 as a pro-growth factor, the MRrhCOL17 MNs achieved environment-responsive IGF-1 release and post-treatment hair coverage of 83% in the reported experimental model [1]. Sun et al. engineered temperature-induced platelet-rich plasma (PRP) gel-encapsulated separable MNs that sustained the release of VEGF, PDGF, and TGF-β for 4–6 days and significantly enhanced regrowth in mouse models [106]. Suo et al. combined PRP lysate with HA MNs (PL-MN), delivering growth factors directly with minimal discomfort, upregulating Ki67 and CD31 to promote proliferation and migration, accelerating anagen entry [107]. Xing et al. devised two recombinant COL17-based MN systems that load IGF-1, promote neovascularization, alleviate inflammation, and accelerate regrowth [108,109]. MN-mediated growth factor delivery enables localized and sustained exposure, and may support complementary biological effects when multiple growth-related signals are delivered together. However, these improvements should not be interpreted as true pharmacological synergy unless they exceed additive responses in appropriately controlled studies (Fig. 4C) [[106], [107], [108]].

### 3.4 Cell therapy strategies

#### 3.4.1 Stem cells and extracellular vesicles in hair regeneration

Stem cells and extracellular vesicles (EVs) provide novel avenues for alopecia therapy, but their clinical translation remains constrained by product heterogeneity, manufacturing reproducibility, and potency standardization [[110], [111], [112]]. Mesenchymal stem cells (MSCs) and their EVs promote repair and regeneration, modulating the niche, activating follicular stem cells, and supporting growth [112]. EVs—particularly exosomes—carry bioactive molecules to facilitate regeneration [112]. Traditional cell injections and exosome delivery face challenges in viability, yield, and targeting [111,112]. Liu et al. reported that intracellular vesicles (IVs) have higher yields with comparable bioactivity, offering an alternative source [111].

#### 3.4.2 MN delivery of cell-derived products

MNs are potentially useful for minimally invasive localized delivery of cell products [[110], [111], [112]]. MSC-derived exosomes were incorporated into separable keratin MNs to enable sustained delivery of follicular stem-cell activators, thereby enhancing regrowth [27]. Jing et al. developed MN-mediated delivery of hypoxia-induced EVs encapsulating selenium nanoparticles (Se-HEVs-AMN), improving the niche by modulating inflammation, promoting angiogenesis, scavenging ROS, and countering androgens, thereby increasing hair density and follicle diameter [110]. Liu et al. created MNs carrying magnetic dental pulp stem-cell IVs (HTMI-MN) using Tremella polysaccharide and HA matrices for multi-component AGA intervention; magnetic IVs enhanced angiogenesis and protected dermal papilla cells from apoptosis, while Tremella polysaccharide promoted macrophage polarization to an anti-inflammatory phenotype [111]. Although these components may have complementary effects, quantitative evidence is required to determine whether their combined effect reflects true synergy rather than additive benefit. In vitro, these systems showed strong pro-angiogenic and protective effects; in mouse models, MN-treated animals exhibited accelerated regrowth [110,111]. Exosome-based MN patches are highlighted as promising for non-scarring alopecia (Fig. 4D) [112]. Despite their regenerative potential, EV- and IV-based MN systems remain difficult to standardize. Vesicle composition depends on cell source, culture conditions, isolation method, particle heterogeneity, and storage protocol, making cross-study comparison and potency definition challenging. Their long-term biodistribution, immunological safety, and potential pro-proliferative risks also require more rigorous investigation.

### 3.5 Collagen and peptide strategies

#### 3.5.1 Roles of collagen and peptides in follicular structure

Collagen is a principal component of skin and follicular architecture, crucial for follicle integrity and function [108,113]. Collagen XVII (COL17) regulates follicular aging, with decreased expression associated with hair loss [108]. Marine-derived collagen peptides have shown pro-growth potential [113], supporting adhesion, proliferation, and stem-cell activity; specific peptide motifs modulate signaling to drive telogen-to-anagen transition [113]. Large molecular size limits skin penetration via conventional topicals [113].

#### 3.5.2 MN delivery of collagen and peptides

MN delivery of collagen and bioactives may help remodel the follicular microenvironment. However, such remodeling is unlikely to be driven by matrix supplementation alone and should be interpreted together with anti-inflammatory, angiogenic, and follicular-targeting effects. COL17 MNs leverage both IGF-1's pro-growth activity and COL17's niche modulation, thereby maintaining stem-cell homeostasis, preventing miniaturization, and supporting regeneration [1]. Zheng et al. designed dissolvable MNs based on marine collagen peptides (GSCPs-MNs) for AGA, mitigating inflammation and promoting collagen formation in vivo, upregulating VEGF and CD31 while downregulating TNF-α, IL-1β, and MDA to alleviate inflammation/oxidative stress and support angiogenesis/follicular function [113]. Xing et al. developed recombinant COL17 MNs with microenvironment responsiveness and low-temperature photothermal effects to control IGF-1 release, achieving superior hair-regeneration outcomes compared with minoxidil in the tested AGA mouse models [108,109]. Hong et al. introduced HAMA/HA hydrogel MNs loading *Platycladus orientalis* extract (PO-ex MN), which scavenged ROS to improve proliferation conditions and activated Wnt/β-catenin signaling via MN-induced mechanical cues to promote follicular growth [114]. MNs effectively bypass the skin barrier to deliver collagen- and peptide-based payloads to follicular regions, contributing to improved hair-regeneration outcomes in preclinical models reported (Fig. 4E) [108,109,113,114]. For collagen- and peptide-based MNs, improved hair growth may arise from combined matrix support, growth-factor signaling, anti-inflammatory effects, and enhanced follicular deposition. However, these multi-mechanistic benefits should not be automatically interpreted as synergy without single-component comparisons and quantitative interaction analysis. In addition, peptide source, molecular weight distribution, degradation behavior, and immunogenicity should be considered when evaluating translational potential.

Taken together, MN-mediated antioxidative, pro-angiogenic, growth factor-based, cell-derived, and collagen/peptide-based strategies provide multiple routes to remodel the perifollicular microenvironment. However, most reported systems demonstrate multi-pathway modulation rather than quantitatively proven synergy. Improved hair-regeneration outcomes may reflect enhanced local deposition, prolonged exposure, mechanical stimulation, additive biological effects, or complementary regulation of pathways. Therefore, future studies should include relevant single-component controls, dose–response comparisons, and quantitative interaction analysis to distinguish additive benefits from true synergistic effects.

## 4 Design and optimization of multi-drug combination delivery

### 4.1 Principles of combination delivery and potential synergy

Multi-drug or multi-agent MN systems combine therapeutic components with complementary mechanisms to enhance hair-regeneration outcomes [[115], [116], [117]]. Given the multifactorial pathogenesis of AGA, monotherapy may be insufficient for patients whose disease involves concurrent androgen signaling, oxidative stress, inflammation, impaired angiogenesis, and follicular stem-cell dysfunction [104]. However, co-delivery should not be automatically equated with true pharmacological synergy. In this review, improved outcomes from multi-agent MN systems are interpreted as additive, complementary, or potentially synergistic depending on whether the combined effect exceeds the expected additive response. MN-mediated combination delivery integrates multiple functional actives within a single platform for localized delivery, enabling coordinated modulation of oxidative stress, vasculature, signaling pathways, and hormonal axes [116,117]. Examples include pairing antioxidants with pro-angiogenic factors to restore redox balance and microvascular support simultaneously, or combining growth-promoting agents with anti-androgens to both stimulate follicular activity and reduce androgenic injury [115,116]. Lin et al. showed that dissolvable MNs co-delivering rapamycin and epigallocatechin gallate (EGCG) nanoparticles significantly improved regrowth beyond single-agent delivery [115], supporting a potentially synergistic interaction that would be strengthened by quantitative combination analysis. Programming differential release kinetics further optimizes temporal and spatial control of multi-agent delivery [117].

### 4.2 Materials and structural designs for multi-agent delivery

Carriers such as liposomes, cubosomes, polymeric nanoparticles, and microspheres can encapsulate diverse actives, protect stability, and enable controlled release [3,21]. Prabahar et al. combined β-sitosterol-loaded nanostructured lipid carriers with MNs (NLC-MNs), markedly increasing transdermal flux relative to the optimized NLC formulation alone [3]. Layered MNs enable sequence-specific release, while tip-loading ensures proximal delivery to follicles [21,117]. Zhang et al. developed composite MNs incorporating Cu/Zn co-doped mesoporous silica nanoparticles (ZCQ/MN) that release quercetin, Cu2+, and Zn2+, forming a multi-component combination platform that coordinates antioxidative, anti-inflammatory, pro-angiogenic, and follicle-activating effects to promote follicular regeneration [116]. Geometry (length, density, shape) should be tailored to drug properties and scalp anatomy [35,118].

### 4.3 Drug combinations: additive, complementary, and synergistic effects

4.3.1 Minoxidil and finasteride serve as cornerstone therapies for androgenetic alopecia (AGA), with markedly complementary mechanisms of action that provide a rational basis for combination treatment [[119], [120], [121]] Minoxidil, a potassium channel opener, promotes hair growth through multiple pathways: it directly stimulates the proliferation of dermal papilla cells and prolongs the anagen phase; acts as a vasodilator to enhance local scalp microcirculation and follicular nutrient supply; exerts anti-inflammatory effects; and activates the Wnt/β-catenin signaling pathway, which is crucial for hair follicle stem cell activation and regeneration [2,122]. Studies demonstrate that minoxidil upregulates vascular endothelial growth factor (VEGF) expression in hair follicles, promoting perifollicular angiogenesis and thereby improving oxygen and nutrient delivery [123].

In contrast, finasteride intervenes in the pathophysiology of AGA primarily through hormonal modulation [10,124]. As a type II 5α-reductase inhibitor, it blocks the conversion of testosterone to the potent androgen dihydrotestosterone (DHT), effectively reducing both scalp tissue and serum DHT levels [11,121,125]. DHT is a key driver of AGA: upon binding to androgen receptors in hair follicles, it shortens the anagen phase, prolongs the telogen phase, and progressively induces follicular miniaturization [10,126]. By lowering DHT levels, finasteride effectively halts this miniaturization process, helping to maintain normal follicular structure and function [15,124]. Its selective inhibition of the type II 5α-reductase isoenzyme significantly reduces DHT-related adverse effects on hair follicles while preserving adequate systemic androgen levels [15].

The mechanisms of these two drugs complement each other on several levels: minoxidil primarily promotes hair growth and improves the follicular microenvironment, whereas finasteride suppresses the underlying hormonal insult [10,13,127]. A temporal difference is also evident: finasteride typically requires 3–6 months to produce noticeable effects, as it works by gradually reducing DHT and reversing miniaturization, whereas minoxidil can promote hair growth within a shorter timeframe (2–3 months) [17,122]. This kinetic difference enables combination therapy to deliver early clinical improvement while laying the foundation for long-term efficacy. At the molecular level, minoxidil-mediated activation of the Wnt/β-catenin pathway and finasteride-mediated suppression of androgen signaling may converge on follicular stem-cell regulation, thereby supporting follicular regeneration and maintenance [123,128]. From a pharmacokinetic perspective, the combination of topical minoxidil and systemic (or topical) finasteride also offers a more comprehensive therapeutic option for patients suboptimally responsive to either agent alone [127,129].

Substantial clinical evidence supports the combined use of minoxidil and finasteride for AGA, with studies employing various designs, populations, and assessment metrics consistently demonstrating its superiority [13,127,130,131].

In male AGA patients, clinical studies, including randomized controlled evidence, have confirmed significant benefits of combination therapy. A randomized, double-blind, controlled trial by Suchonwanit et al. compared a combined topical solution containing 0.25% finasteride and 3% minoxidil with 3% minoxidil monotherapy [130]. Results at 24 weeks showed that the combination solution was significantly superior to minoxidil alone across hair density, hair shaft diameter, and global photographic assessment (all P < 0.05) [130]. Notably, approximately 90% of patients receiving the combination solution experienced moderate to marked improvement, with only a minimal reduction in plasma DHT (∼5%) and no reported systemic adverse events [130]. This clinical finding suggests that topical combination therapy may offer efficacy comparable to oral finasteride while significantly reducing systemic risk [130,132].

Clinical evidence in female AGA patients is more limited than in male AGA, but available studies also support potential efficacy in selected groups. While oral finasteride is generally not recommended for women of childbearing potential, the combination of topical finasteride with minoxidil shows promise [133,134]. A systematic review of clinical studies evaluating oral or topical finasteride, either as monotherapy or in combination, for pre- and postmenopausal women with female pattern hair loss (FPHL) found that 10 studies reported high rates of hair restoration in women taking finasteride [134]. Based on the outcomes, oral finasteride at 5 mg/day may be an effective and safe treatment for women with FPHL, particularly when combined with other agents like topical estradiol and minoxidil [134]. The review also concluded that topical finasteride was more effective than other topical formulations for treating alopecia [134]. Nevertheless, evidence for finasteride in female AGA remains more heterogeneous than in male AGA, and interpretation should account for reproductive status, dose selection, treatment duration, and the limited number of well-controlled studies.

A single-masked randomized clinical trial by Sadeghzadeh-Bazargan et al. compared two combination regimens: topical minoxidil plus oral spironolactone versus topical minoxidil plus oral finasteride [135]. The study enrolled 60 female AGA patients, including both FPHL and male-pattern (MPHL) phenotypes [135]. Results at 4 months revealed significant differences between the two groups in treatment response (physician satisfaction), hair density, and alopecia severity [135]. The minoxidil-spironolactone group had a 6.7% non-response rate, compared to 16.7% in the minoxidil-finasteride group; 56.7% of patients in the minoxidil-spironolactone group achieved an excellent treatment outcome, versus 0% in the minoxidil-finasteride group, a statistically significant difference (p = 0.01) [135]. Regarding patient satisfaction, minoxidil-spironolactone treatment was significantly superior to minoxidil-finasteride in improving hair density and alopecia severity (p = 0.01) [135]. This indicates that in female AGA patients, particularly those with MPHL patterns, combining minoxidil with an antiandrogen like spironolactone may be more effective than combining it with finasteride [135].

Long-term efficacy and safety data further support the value of combination therapy. Multiple studies indicate that combination treatment not only yields significant short-term results but also maintains efficacy over time with a favorable safety profile [13,127]. A network meta-analysis by Gupta et al. comparing six common non-surgical AGA treatments found combination therapy to be comparable or superior to other effective interventions in terms of mean change in hair count [136]. Although the most drug-related adverse events were reported for minoxidil 5% and minoxidil 2% (n = 45 and n = 23, respectively), the overall safety of combination therapy remained acceptable [136]. Importantly, the use of topical finasteride formulations may further mitigate systemic adverse effects, improving the safety of combination regimens [16,130,132].

Microneedling (MN), an emerging physical transdermal delivery enhancement technology, can be combined with minoxidil and finasteride to enhance local drug permeation and provide adjunctive mechanical stimulation, offering an additional strategy for AGA management [[137], [138], [139], [140]]. By creating microchannels in the skin, MNs significantly enhance drug permeation and increase bioavailability at the follicular target site [137,141]. However, the magnitude of this enhancement may vary substantially with needle length, rolling pressure, treatment interval, micropore closure kinetics, and scalp contact conditions, thereby complicating direct comparisons across clinical protocols. This enhancement is particularly valuable for both minoxidil, which has a relatively high molecular weight limiting its skin penetration, and finasteride, which suffers from poor aqueous solubility and low local bioavailability [138,141]. MN technology effectively overcomes these barriers, enabling deeper drug penetration into the follicular region and thereby improving treatment outcomes [137].

Mechanistically, combining MN with pharmacological agents offers multiple advantages. Firstly, the microchannels created by MNs not only facilitate drug delivery but also stimulate a wound-healing response by recruiting various growth factors and cytokines, such as platelet-derived growth factor (PDGF), transforming growth factor-β (TGF-β), and epidermal growth factor (EGF) [142,143]. These factors themselves promote hair follicle stem cell activation and prolong anagen, complementing the effects of minoxidil and finasteride [123,142]. Secondly, MN treatment may improve local scalp blood circulation and complement the vasodilatory action of minoxidil, collectively enhancing nutrient and oxygen supply to the follicles [137]. Furthermore, the mild inflammatory response induced by MN may help remodel the perifollicular microenvironment, making it more conducive to hair growth [143].

In summary, the integration of MN with minoxidil and/or finasteride can enhance treatment efficacy through improved local delivery, adjunctive mechanical stimulation, and complementary pharmacological mechanisms [137,138]. Clinical studies have confirmed the significant advantages of this combined strategy, including greater hair density, increased hair shaft diameter, and higher patient satisfaction [137]. With ongoing advancements and innovation in MN technology, this combination strategy is poised to become a mainstay in AGA management, particularly for patients with suboptimal response to conventional therapies or intolerance to systemic adverse effects [137,138,141].

**Fig. 5:**
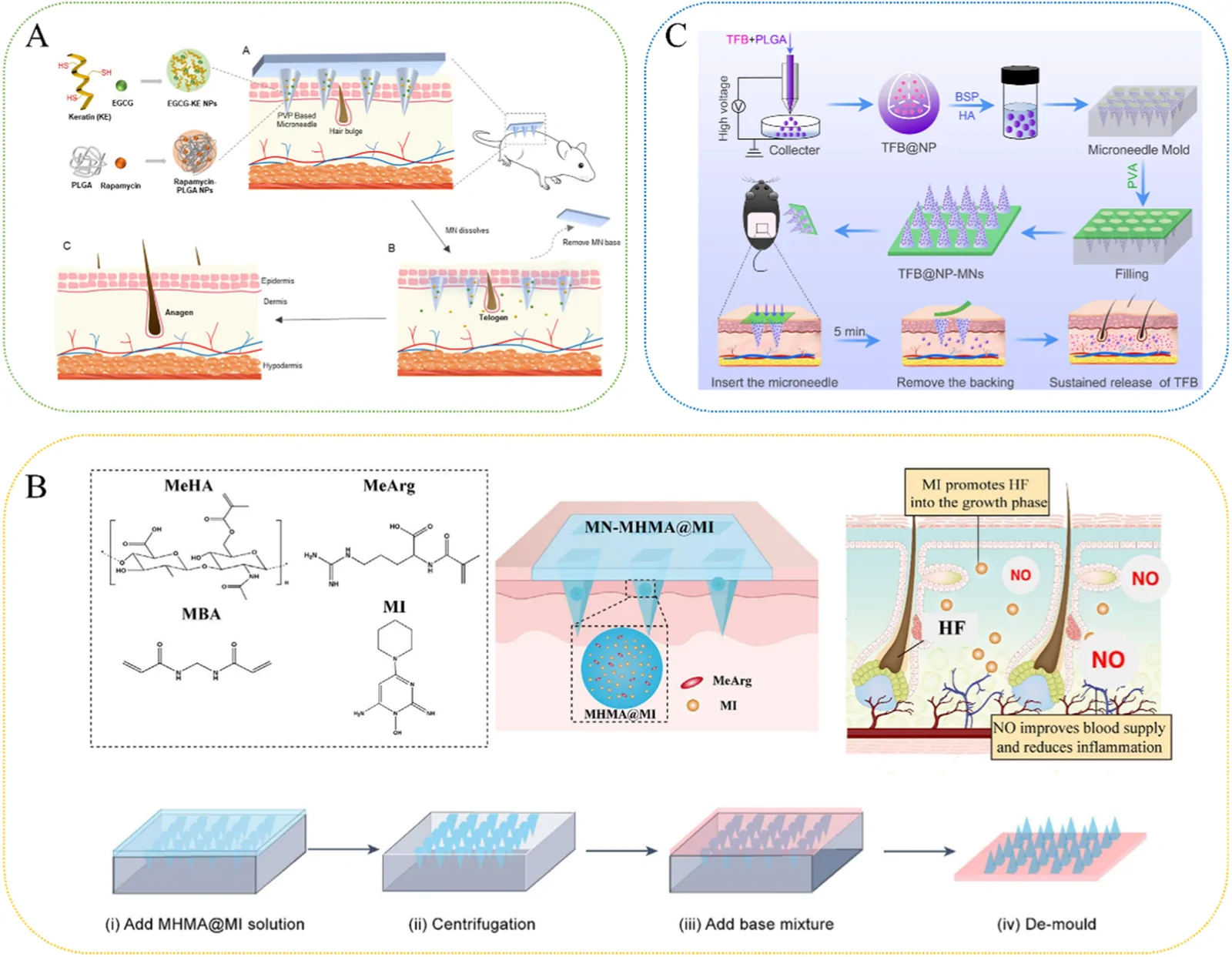
**Microneedle-based delivery systems enabling synergy between natural products and synthetic drugs.** (A) Microneedles co-loaded with rapamycin and epigallocatechin gallate nanoparticles promote hair regeneration through synergistic inhibition of the mTOR pathway and scavenging of oxidative stress. Reproduced with permission from Ref. [115]. Copyright 2022, MDPI. (B) Microneedles co-delivering a nitric oxide donor and minoxidil leverage the synergistic effects of vasodilation, anti-inflammatory activity, and hair follicle stimulation to accelerate the anagen transition. Reproduced with permission from Ref. [144]. Copyright 2025, Wiley. (C) Nanocomposite microneedles based on *Bletilla striata* polysaccharide and tofacitinib utilize the adhesive and sustained-release properties of the polysaccharide for prolonged drug delivery, promoting hair growth through combined mechanical stimulation and anti-inflammatory action. Reproduced with permission from Ref. [76]. Copyright 2025, Elsevier.

4.3.2 Antioxidants + Pro-Angiogenic Factors Co-delivering antioxidants with vasodilators or pro-angiogenic factors addresses the vicious cycle wherein ROS damage both follicular cells and microvasculature [43,101]. In an AGA mouse model, Yang et al. combined a polydopamine–quercetin liposomal system (PDA@QLipo) with roller MNs to remodel the niche, achieving 92.5% coverage in AGA mice—surpassing minoxidil (87.8%)—with lower dosing frequency [43]. Although encouraging, this comparison was obtained in a preclinical model, and the relatively small numerical difference in coverage should be interpreted with caution until validated against standardized dosing schedules and clinically relevant endpoints. Cu/Zn co-doped quercetin systems concurrently suppressed DHT, mitigated inflammation, promoted angiogenesis, and activated follicular stem cells [116].
4.3.3 Combination delivery of growth factors (VEGF, IGF-1, PDGF) with signaling modulators can support coordinated regulation of follicular development and cycle transitions [19,106]. Ding et al.’s hydrogel MNs co-delivering VEGF and ritlecitinib/PHAs nanoparticles promoted angiogenesis, improved the immune microenvironment, and enhanced proliferation, rapidly initiating anagen and improving hair quality and coverage in preclinical AGA models [19]. PRP-based MNs sustained multi-factor release in response to mechanical stimulation and markedly promoted regrowth in the reported animal models [106].
4.3.4 Natural Products + Synthetic Drugs Natural products (plant extracts, traditional medicines) often provide multi-target activity with lower toxicity; combining them with targeted synthetic drugs yields complementary effects [76,115]. However, for MN-based combination delivery, the complex composition, batch-to-batch variability, uncertain active components, and potential interactions with synthetic drugs remain important formulation and regulatory concerns. In preclinical evaluation, Lin et al. demonstrated that rapamycin + EGCG nanoparticles in dissolvable MNs jointly modulated mTOR and redox pathways and enhanced regrowth beyond single-agent delivery, indicating a potentially synergistic interaction (Fig. 5A) [115]. In preclinical models, Wang et al. co-loaded a nitric oxide donor and minoxidil into MNs (MHMA-MNs), thereby improving vascular supply and reducing inflammation to facilitate anagen entry (Fig. 5B) [144]. In preclinical models, Zhao et al. incorporated tofacitinib nanoparticles into *Bletilla striata* polysaccharide-based dissolvable MNs (TFB@NP-MNs) for 48-h sustained release; mechanical stimulation combined with sustained pharmacology promoted regrowth (Fig. 5C) [76]. MN-based co-delivery of minoxidil with anti-androgens, anti-inflammatories, or growth factors may support broader modulation of the perifollicular microenvironment through complementary mechanisms [125,131].

Overall, current evidence supports the therapeutic rationale for MN-mediated combination delivery in AGA, particularly when paired agents target distinct yet related pathological processes, such as androgen signaling, vascular insufficiency, inflammation, oxidative stress, and follicular stem-cell dysfunction. Clinically, minoxidil–finasteride combinations have demonstrated clear advantages over monotherapy in selected patient groups. In contrast, many emerging MN-based multi-agent systems are best interpreted as complementary or potentially synergistic strategies at the preclinical stage. Future optimization should focus on dose ratio, release sequence, follicular deposition, and appropriate single-agent comparisons to clarify the therapeutic interaction underlying each combination.

## 5 Core features of intelligent stimulus-responsive MN systems

### 5.1 ROS-responsive MNs

In preclinical AGA studies, ROS-responsive MNs exploit excessive ROS in AGA niches as triggers for on-demand release [[101], [102], [103]]. In preclinical models, ceria nanozyme MNs (Ce-MNs) with catalase- and SOD-like activity efficiently cleared peri-follicular ROS [101]. Pt nanozyme dissolvable MNs (Pt-MNs) reduced ROS to O2, alleviating oxidative stress and enhancing local oxygenation to support stem-cell differentiation [103]. CuxO nanozyme-functionalized MNs (CuxO-MNs) likewise scavenged ROS and promoted telogen-to-anagen transition [102]. These preclinical platforms enable targeted recognition of pathological microenvironments and precise antioxidative action (Fig. 7A) [[101], [102], [103]]. Nevertheless, ROS-responsive systems require careful calibration, because physiological ROS also participate in follicular signaling and tissue repair; moreover, the long-term scalp retention, biodegradation, and immunological safety of nanozyme components remain important translational questions.

### 5.2 Enzyme-responsive MNs

**Fig. 6:**
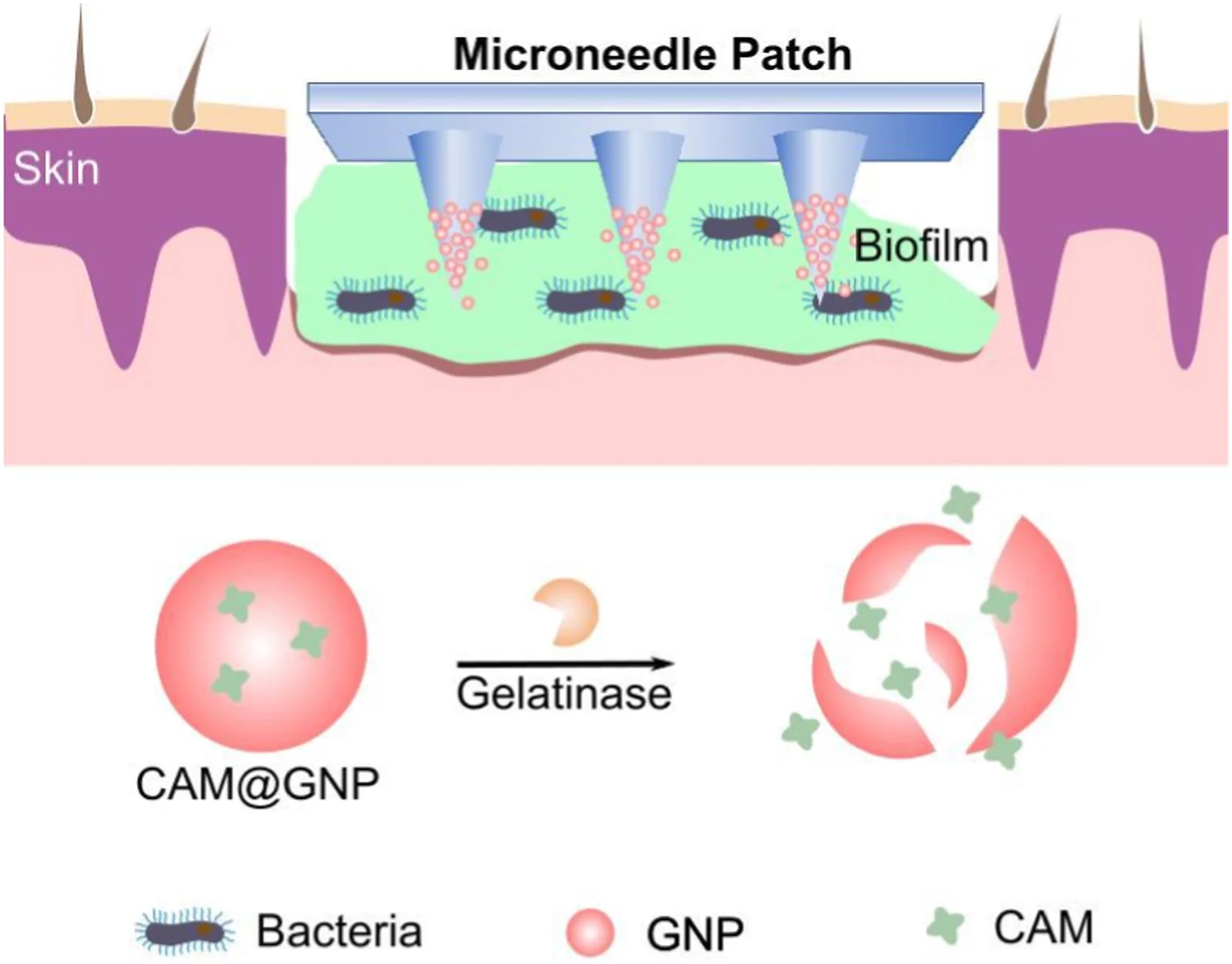
**Schematic illustration of biofilm treatment mediated by microneedles.** The microneedle array penetrates the biofilm and dissolves, releasing gelatin nanoparticles (GNPs) loaded with chloramphenicol (CAM). These GNPs respond specifically to gelatinase secreted by metabolically active bacteria, enabling targeted antibiotic release at the site of infection to eliminate pathogenic bacteria within the biofilm while minimizing off-target effects. Reproduced with permission from Ref. [145]. Copyright 2019, American Chemical Society.

Enzyme-responsive MNs harness spatiotemporal heterogeneity of enzymes (MMPs, gelatinases MMP-2/9, elastase, lysozyme, glucose oxidase) and H2O2 generation to trigger release via carrier depolymerization, pore formation, or bond cleavage [145,146]. In preclinical disease models of non-alopecia, Xu et al. developed chloramphenicol–gelatin nanoparticles (CAM@GNPs) integrated into MNs for biofilm infections; the particles selectively disassembled in gelatinase-rich niches to release the drug (Fig. 6) [145]. Singh et al. designed dissolvable polymer MNs that integrate glucose oxidase with NIR-responsive CuS nanozymes to enable cascade reactions and light-controlled ROS generation in cancer [146]. While primarily applied in infection/oncology, these principles provide a preclinical design rationale that could be adapted to alopecia—for example, by designing MNs sensitive to follicular enzymes, including 5α-reductase, for targeted anti-alopecia release [104]. However, this remains a conceptual extrapolation, and practical implementation would require validated enzyme gradients in human scalp, sufficient substrate specificity, and avoidance of nonspecific release in surrounding skin.

**Fig. 7:**
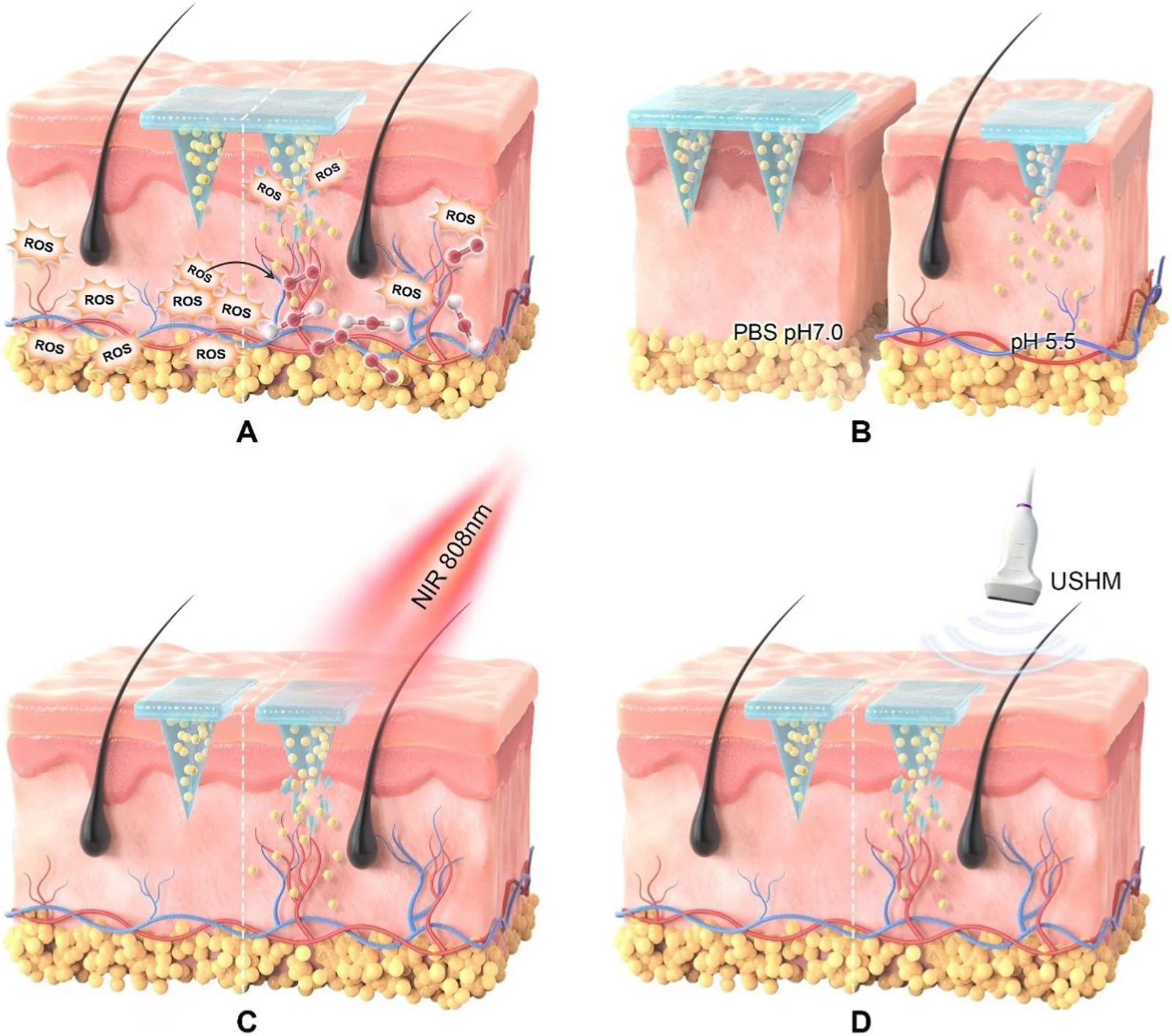
**Schematic illustration of the mechanisms of intelligent stimulus-responsive microneedle systems.** (A) **ROS-Responsive**: The system releases encapsulated nanozymes (e.g., CeNZs, PtNZs) when excessive reactive oxygen species (ROS) accumulate in the perifollicular microenvironment, enabling in situ ROS scavenging and microenvironmental repair. (B) **pH-Responsive**: Sensing changes in tissue pH (e.g., from physiological pH 7.4 to the weakly acidic milieu of inflamed or follicular regions), the system dissolves or swells at a specific pH, achieving on-demand and targeted drug release. (C) **Near-Infrared (NIR) Light-Responsive**: Upon external NIR irradiation (e.g., at 808 nm), photothermal agents (e.g., gold nanorods, polydopamine) within the system generate heat, softening or degrading the microneedle matrix to enable controlled drug release. (D) **Ultrasound-Responsive**: Externally applied ultrasound generates cavitation and mechanical forces within the microneedles or skin tissue, enhancing drug diffusion and tissue permeation to promote drug delivery synergistically.

### 5.3 pH-responsive MNs

In preclinical hair-regeneration studies, pH-responsive MNs modulate release in response to local pH gradients within the skin and follicles [104,147]. In a preclinical AGA setting, Lv et al. designed dual-functional HA MNs that integrate glycyrrhizin–dutasteride (GL-DUT) micelles and mesoporous polydopamine nanoparticles (MPDA), achieving acid-triggered release of combined anti-androgenic and antioxidative modulation via 5α-reductase inhibition and ROS scavenging [104]. In preclinical hair-regeneration models, Chen et al. developed a “seed–soil” dual-target nanotherapeutic that simultaneously alleviates oxidative stress and activates autophagy to promote hair regeneration, leveraging pH-responsive release within follicular niches (Fig. 7B) [147].

### 5.4 Temperature-responsive MNs

Temperature-responsive MNs exploit thermally induced polymer phase transitions or gelation for controlled release [98,148]. In a preclinical PRP-MN system, Sun et al. built a dual-functional PRP MN system whose temperature-induced gelation and reservoir properties enabled rapid gel formation at skin temperature and sustained release of VEGF, PDGF, and TGF-β; interpenetration with photo-crosslinked GelMA increased single-MN mechanical strength (1.21 N) and achieved 4–6 days of controlled growth factor release [106]. In experimental evaluations, Zhang et al. developed hexagonal nanocomposite MNs using poly(acrylic acid)–N-hydroxysuccinimide hydrogels, exhibiting temperature responsiveness and strong adhesion in moist environments [148]. Low-temperature photothermal effects have also been combined to fine-tune release (e.g., recombinant COL17 systems) [108].

### 5.5 Other stimuli-responsive systems

Magnetic-responsive MNs use external fields to trigger release or enhance transport; in preclinical studies, Liu et al.’s magnetic dental pulp IV-loaded MNs (HTMI-MN) demonstrated magnetically modulated delivery [111]. Light-responsive MNs utilize photothermal/photodynamic effects under NIR irradiation for remote-controlled, on-demand release, suitable for hair-bearing scalp regions given NIR tissue penetration (Fig. 7C) [94,149]. However, their scalp application requires careful control of irradiation dose, photothermal conversion efficiency, and exposure duration, because hair coverage, skin pigmentation, and local heat accumulation may affect both release reproducibility and safety. Electrical stimulation can modulate release via electrophoresis/electroosmosis, enhancing transport of charged species (e.g., HA ions, rhodamine B) [150]. Self-powered triboelectric MN platforms have also been demonstrated in preclinical wound-healing models; for example, tongue-prick-inspired, angle-adjustable MNs integrated with triboelectric nanogenerators generated motion-driven electrical stimulation and improved scarless tissue repair, supporting the feasibility of combining mechanical anchoring, microfluidic transport, and electrical actuation within a single MN platform [151]. Ultrasound-responsive systems facilitate permeation and release, as demonstrated by USHM arrays for finasteride (Fig. 7D) [152]. In preclinical AGA models, Wang et al. developed androgen receptor-targeting PROTAC MNs (PROTAC-MNs), achieving localized AR degradation and effective single-application control of AGA and recurrence [18]. Despite this promising result, AR degradation is a potent and mechanism-specific intervention; therefore, local selectivity, reversibility, effects on non-follicular androgen-responsive cells, and long-term recurrence after repeated hair cycles require further evaluation. Machine-learning-guided MN composition optimization has also been explored as an engineering optimization approach to enhance Wnt/β-catenin activation and improve hair regrowth [153].

## 6 Closed-loop systems and real-time monitoring

### 6.1 Principles of closed-loop control in intelligent microneedle systems

**Fig. 8:**
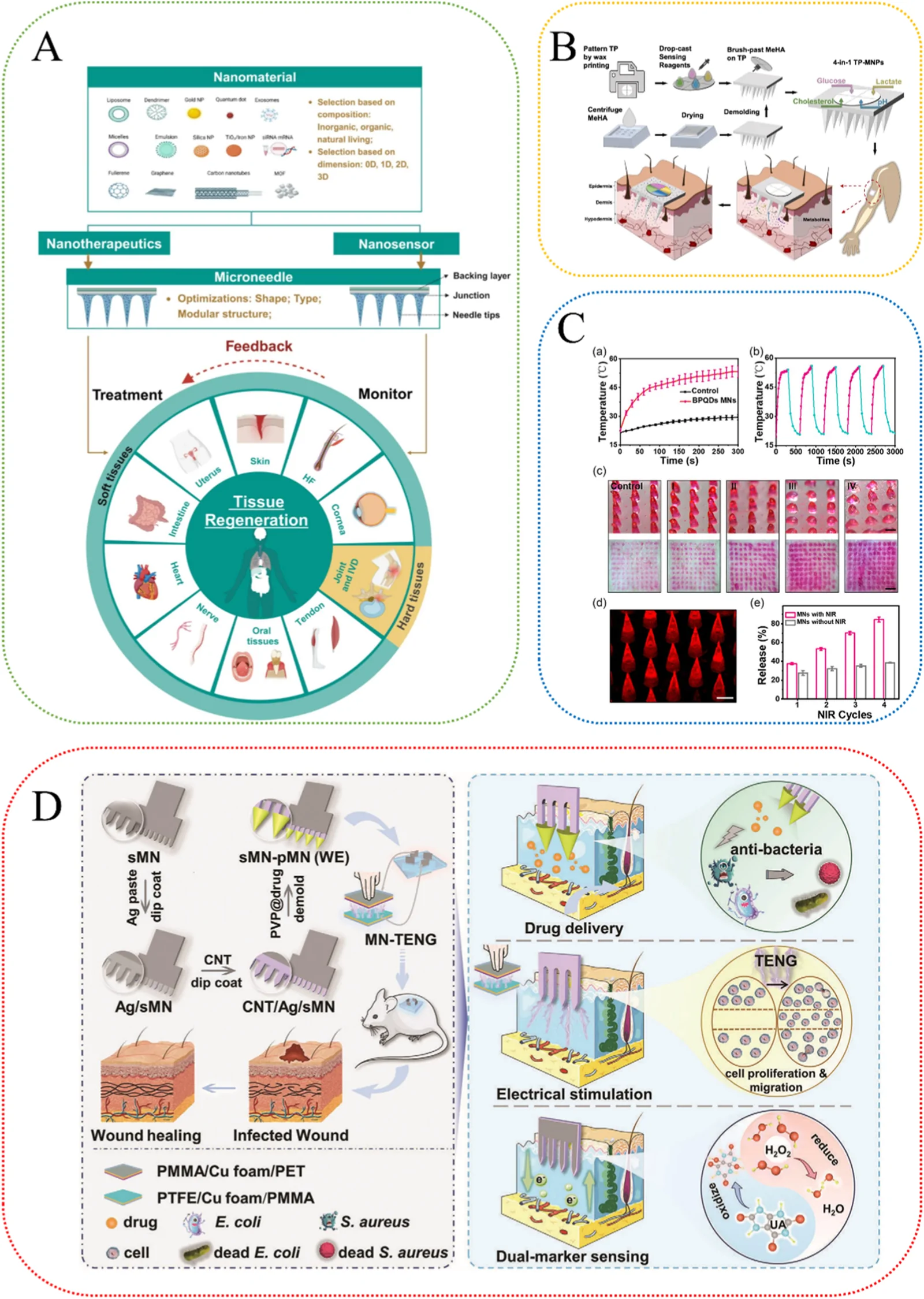
**Schematic illustration of intelligent microneedle platforms for real-time monitoring and closed-loop therapy.** (A) Overview of nanomaterial-integrated microneedle systems combining nanotherapeutics and nanosensors for tissue regeneration, highlighting the dual functions of treatment and monitoring [156]. Copyright 2025, Journal of Nanobiotechnology. (B) Hydrogel microneedle patch for interstitial fluid extraction and colorimetric detection of multiple biomarkers (e.g., glucose, lactate, cholesterol, pH). The microneedles swell upon insertion into the skin, absorb ISF, and enable on-device colorimetric readout [157]. Copyright 2022, Elsevier B.V. (C) Near-infrared (NIR)- responsive separable microneedles for on-demand drug release. Black phosphorus quantum dots embedded in the needle tips generate heat upon NIR irradiation, thereby melting and releasing the drug [158]. Copyright 2024, Wiley-VCH GmbH. (D) Fully integrated closed-loop microneedle patch with triboelectric nanogenerator (TENG) for self-powered actuation and electrochemical dual-marker sensing. The device consists of drug-loaded microneedles, Ag/CNT-coated sensing electrodes, and a TENG backing layer that converts mechanical energy into electrical stimulation to trigger drug release while simultaneously monitoring wound biomarkers (H_2_O_2_ and uric acid) [159]. Copyright 2024, Wiley-VCH GmbH.

As a frontier technology in hair regeneration, intelligent microneedle (MN) systems represent a conceptual and preclinical framework for applying precision medicine to alopecia therapy through closed-loop control [138,154]. A closed-loop system continuously monitors physiological parameters, automatically adjusts therapeutic variables, and optimizes regimens based on feedback [138,154]. In hair regeneration, such systems typically comprise three core components: a sensing module, a processing module, and an actuation (execution) module [138]. The sensing module continuously tracks scalp microenvironment parameters such as pH, temperature, humidity, inflammatory mediators, and follicle status; the processing module analyzes these inputs using pre-defined algorithms to make decisions; and the actuation module modulates drug release, MN stimulation intensity, and other treatment parameters according to the decisions [138,154]. This iterative cycle of sensing–analysis–adjustment constitutes the foundation of closed-loop control, with the potential to support dynamic adaptation to changes in scalp state and individualized precision therapy [138]. Recent advances have demonstrated that integrating nanosensors into MN platforms enables their use as smart wearable devices for real-time monitoring of damaged tissues via interstitial fluid (ISF) extraction and biomarker sensing, thereby establishing the technological foundation for closed-loop therapeutic systems [155] (Fig. 8A).

The sensing module integrates advanced biosensing technologies that capture key scalp microenvironment data noninvasively or minimally invasively [138,154]. Microfluidic sensors can quantify biomarkers in interstitial fluid—e.g., dihydrotestosterone (DHT), inflammatory cytokines (IL-6, TNF-α), and growth factors (VEGF, IGF-1)—that directly reflect follicular growth status and pathology [154]. However, for AGA-oriented monitoring, the clinical validity of these ISF biomarkers remains to be established, particularly with respect to their correlation with follicular activity, scalp tissue levels, and long-term hair-density outcomes. Optical sensors assess follicle density, blood perfusion, and hair-cycle distribution by analyzing reflected/scattered spectra from the scalp [154,160]. Electrochemical sensors measure pH, conductivity, and related physicochemical parameters closely tied to scalp health [138]. For instance, microneedle-based electrochemical sensors utilizing organic electrochemical transistors (OECTs) have been developed for continuous glucose monitoring, demonstrating the feasibility of integrating enzymatic sensing elements with microneedle platforms for real-time biomarker detection [159,161] (Fig. 8D illustrates a typical configuration of such integrated electrochemical sensing microneedles). Beyond enzyme-based glucose sensing, integrated hydrogel MN aptamer sensors have recently been developed for continuous insulin monitoring in interstitial fluid, using swollen hydrogel microneedles to access ISF and aptamer-functionalized electrochemical electrodes to detect low-abundance bioanalytes in real time [162]. Similarly, microneedle electrodes coated with conductive nanomaterials, such as carbon nanotubes or metal nanoparticles, enable sensitive detection of various analytes in tissue fluids [163]. Miniaturization and integration allow these sensors to be embedded within MN systems for real-time, multi-site monitoring of the scalp microenvironment [138,154].

The processing module functions as the “brain” of the system, employing advanced algorithms and artificial intelligence to analyze sensed data and make treatment decisions [138]. Typically built on machine learning or deep learning, these models are expected to learn patterns linking scalp parameters to therapeutic outcomes from future large-scale clinical datasets [138,154]. For example, the system can infer follicular response profiles across varying DHT levels or determine optimal drug-release rates under different inflammatory states [154]. Beyond interpreting current signals, the processing module leverages historical data to predict changes in scalp status trajectory, enabling proactive adjustments rather than purely reactive responses [138]. Advanced modules further incorporate adaptive learning to continuously refine decision rules based on an individual's treatment responses, achieving genuine personalization [138,154]. The integration of artificial intelligence with nanosensor-enabled MN platforms holds particular promise for personalized regenerative medicine, as AI algorithms can process complex, multi-parameter biomarker data to optimize treatment protocols in real time [156]. Nevertheless, AI-guided decision-making will depend on high-quality longitudinal datasets, clinically meaningful outcome labels, model interpretability, and safeguards against inappropriate dose adjustment based on noisy or biased sensor inputs.

The actuation (execution) module serves as the “arm” of the system, implementing parameter changes commanded by the processing module [138,141]. For drug delivery, it can control the release rate, timing, and total dose by adjusting micro-pump pressure, tuning the degradable matrix kinetics, or modulating the applied electric/magnetic fields [138,141]. For MN stimulation, needle length, array density, stimulation frequency, and duration can be varied to optimize biophysical effects [137]. Some advanced actuators enable programmed multi-drug release—e.g., prioritizing anti-inflammatory agents when high inflammation is detected and favoring growth promoters when follicles are in telogen [138]. System performance depends critically on actuator precision and response speed; thus, high-precision microactuators and fast-response materials are typically employed [138,141]. Notably, recent innovations have enabled the development of self-powered actuation systems, such as triboelectric nanogenerators integrated into MN patches, which convert biomechanical energy from joint movement into electrical stimulation to enhance drug delivery and tissue regeneration [164,165]. (Fig. 8D provides a comprehensive illustration of such a closed-loop system integrating self-powered actuation and real-time sensing). For scalp-oriented closed-loop use, however, the intermittency of motion-derived energy, variability of electrical output, long-term mechanical fatigue, and safe dosing of electrical stimulation remain unresolved engineering constraints.

Several key technical challenges confront the practical deployment of closed-loop MN [138,154]. First, sensor stability and specificity: the scalp microenvironment is complex and dynamic, making long-term stable operation and selective detection difficult [154]. In nanosensor-integrated MN platforms, issues such as signal drift in electrochemical sensors and the need for frequent recalibration remain significant hurdles for long-term continuous monitoring [166]. Second, power supply: continuous monitoring and computation require sustained energy, motivating development of efficient, safe, miniaturized power solutions [138]. Emerging approaches include biofuel cells that generate electricity from endogenous substrates such as glucose, offering potential for self-powered sensing systems [167]. Third, biocompatibility and safety: all materials contacting the scalp must be biocompatible and non-sensitizing, avoiding inflammatory or allergic reactions [138,141]. The incorporation of nanomaterials necessitates rigorous long-term toxicological evaluation to ensure their safety profiles, particularly for chronic applications in hair regeneration [156]. Finally, algorithm reliability is critical because decisions directly affect efficacy and safety; ensuring robust, safe decision-making across diverse conditions is central to system design [138,154].

Despite these hurdles, closed-loop control in intelligent MN systems has shown substantial promise mainly in laboratory studies, with limited early clinical exploration [138,154]. For instance, conceptual closed-loop designs could automatically adjust finasteride release rates according to sensed DHT levels to maintain drug concentrations within an optimal therapeutic window [138]. Others tune physical stimulation parameters based on hair-cycle distribution—providing stronger stimulation during anagen and attenuating it during telogen to minimize injury [154,160]. In parallel, wearable MN devices integrating multiplexed sensors have been developed for continuous, real-time monitoring of multiple biomarkers in tissue fluids, demonstrating the feasibility of comprehensive, multi-parameter health tracking that could be adapted for scalp monitoring in AGA therapy [168]. Such innovations not only improve outcomes but also reduce adverse events, pointing to a future direction in AGA therapy [138,154].

Overall, closed-loop control principles in intelligent MN systems integrate advanced sensing, AI-driven analytics, and precise actuation to enable real-time monitoring and dynamic adjustment of AGA therapy [138,154]. This precision-medicine approach can overcome the limitations of one-size-fits-all, deliver truly individualized regimens, improve efficacy, and reduce adverse effects [138,154]. The convergence of nanomaterial-based sensing, flexible electronics, and wireless communication technologies is accelerating the development of next-generation intelligent MN platforms capable of autonomous closed-loop operation [155,156]. With continued technological maturation and clinical validation, closed-loop intelligent MN systems may become future adjunctive tools for AGA care [138,141,154].

### 6.2 Applications of real-time monitoring technologies in hair regeneration with intelligent microneedle systems

Real-time monitoring is the foundation of closed-loop control and among the most innovative aspects of intelligent MN systems for hair regeneration [154,160]. These technologies capture dynamic changes in the scalp microenvironment and follicle status noninvasively or minimally invasively, providing critical data for precision therapy [138,154]. Core applications include follicle status assessment, drug concentration monitoring, treatment efficacy evaluation, and early warning of adverse events [154,160]. Collectively, they enable clinicians and patients to understand therapeutic progress better and promptly adjust regimens to achieve truly individualized treatment [138,160]. The ability to continuously monitor biomarkers in interstitial fluid via MN-based sensors has been extensively demonstrated in other medical contexts, providing a technological foundation for adapting these approaches to hair regeneration [166]. For example, wearable hydrogel MN aptamer sensors have been proposed for real-time insulin monitoring in diabetes management, illustrating how MN-accessed ISF can be coupled with electrochemical molecular recognition for continuous therapeutic monitoring; however, challenges such as low analyte abundance in ISF, sensor drift, and mapping ISF levels to clinically validated plasma biomarkers remain important translational barriers [162].

Follicle status assessment is a central application of real-time monitoring [154,160]. Traditional evaluations rely on clinical observation and dermoscopy, which, although useful, are subjective and lack continuity [154]. Integrated advanced sensing within intelligent MN systems overcomes these limitations by providing objective, continuous data [138,154]. For example, miniaturized optical coherence tomography (OCT) sensors can image follicular structures with high resolution and track real-time changes in size, morphology, and depth [160]. Multispectral imaging quantifies blood perfusion, oxygenation, and metabolic activity by analyzing wavelength-dependent tissue interactions [154,160]. Electrical impedance measurements capture changes in peri-follicular tissue impedance closely associated with hair-cycle phases (anagen, catagen, telogen) [154]. Microneedle-based sensors have also been developed for extracting interstitial fluid from tissues and analyzing its biomarker content, providing a minimally invasive window into the local microenvironment [157]. Hydrogel-based microneedles, in particular, can absorb ISF through swelling, enabling subsequent offline analysis or integrated on-device detection of relevant biomarkers [169,170] (Fig. 8B shows a hydrogel microneedle patch with integrated colorimetric detection for multiple biomarkers). Taken together, these modalities enable a comprehensive assessment to inform therapeutic decisions [138,154,160].

Drug concentration monitoring is another key application [138,141]. In AGA treatment, the local drug concentration at the target site directly determines both efficacy and the risk of adverse effects [141]. Traditional administration struggles to precisely control local exposure, whereas intelligent MN systems can integrate microsensors to measure intradermal drug levels in real time [138]. Electrochemical sensors can quantify minoxidil or finasteride concentrations with high selectivity and sensitivity, distinguishing analytes from interferents [138,141]. In practice, real-time intradermal monitoring of these drugs remains technically challenging because local concentrations may be low, tissue binding can complicate signal interpretation, and sensor calibration in scalp ISF has not been standardized. Fluorescent/optical sensors track labeled drug distribution and metabolism, providing real-time pharmacokinetic data [138]. Advanced approaches include surface-enhanced Raman scattering (SERS)-based microneedle sensors, which enable sensitive and specific detection of drug molecules in dermal interstitial fluid through their unique spectral fingerprints [171]. Colorimetric microneedle sensors incorporating enzyme-linked reactions offer simple, visual readouts of analyte concentrations, potentially enabling patient self-monitoring of drug levels [157] (see Fig. 8B for an example of such a colorimetric sensor platform). Such data help maintain concentrations within the therapeutic window, optimize dosing time and frequency, and support dose individualization based on interpatient metabolic differences [138,141].

Treatment efficacy evaluation is a direct clinical application of real-time monitoring [28,29]. Conventional endpoints (hair counts, shaft diameter) are informative but require prolonged observation [154]. Intelligent MN systems can predict outcomes earlier by tracking microenvironmental changes [154,160]—for instance, monitoring peri-follicular angiogenesis, an early marker of hair growth [123,154]; declines in inflammatory mediators (IL-6, TNF-α) indicative of effective therapy [154]; and increases in growth factors (VEGF, IGF-1, FGF) reflecting follicle activation and regeneration [123,154]. The ability of nanosensor-integrated MN platforms to continuously monitor multiple biomarkers simultaneously provides a comprehensive picture of the regenerative process, enabling early assessment of therapeutic response before clinical changes become apparent [155]. For example, monitoring reactive oxygen species (ROS) levels in the perifollicular microenvironment can indicate the resolution of oxidative stress, a key factor in hair follicle health and regeneration [172]. Early detection of these signals enables regimen adjustments before overt clinical change, improving timeliness and effectiveness [138,154,160].

Adverse-event early warning is an essential safety application [138,141]. While effective, AGA drugs such as minoxidil and finasteride can cause local irritation, contact dermatitis, or sexual side effects [16,124]. By continuously monitoring the scalp microenvironment, intelligent MN systems can detect early warning signs [138]—elevations in IL-1β, IL-6, or histamine suggestive of contact dermatitis [154]; abnormal pH or conductivity shifts indicating irritation [138]; and, for oral finasteride, changes in scalp DHT and sex hormone–related biomarkers signaling potential sexual adverse effects [154]. Wearable microneedle sensor platforms have demonstrated the capacity for real-time detection of inflammatory cytokines and other biomarkers associated with adverse reactions, providing a technological basis for early warning systems in AGA therapy [173]. For instance, electrochemical immunosensors integrated into microneedles can detect picomolar concentrations of pro-inflammatory cytokines such as IL-6 and TNF-α, enabling rapid identification of inflammatory responses [173,174]. Early alerts enable timely interventions (dose adjustment, drug switching, symptomatic treatment), enhancing safety [138,141].

Several technical challenges remain for real-time monitoring in hair regeneration [138,154]. These include miniaturization and integration of multiple sensors within limited scalp real estate [138]; long-term stability and durability in a variable environment [154]; signal drift and calibration requirements for continuous electrochemical sensing [166,175]; data-interpretation complexity, requiring advanced algorithms to extract actionable insights from high-dimensional signals [138,154]; and cost/access barriers that must be addressed for broad clinical adoption [138,160]. Furthermore, the translation of nanosensor-integrated MN platforms from laboratory to clinic requires rigorous validation of their safety, reliability, and performance in human subjects, as well as the development of scalable manufacturing processes [19].

Despite these challenges, the potential is substantial [154,160]. Some intelligent MN systems have been proposed or prototyped for real-time monitoring of follicle status, drug levels, and treatment response [138]. For example, systems developed by Kim et al. integrate multiple microsensors to simultaneously track follicle density, perfusion, and inflammatory mediators, offering comprehensive datasets for AGA management [154]. Others focus on wearable monitors that interoperate with MN platforms to extend monitoring duration and coverage [160]. Recent innovations include fully integrated wearable microneedle array devices for wireless, continuous real-time monitoring of multiple biomarkers in tissue fluids, demonstrating the feasibility of comprehensive, multi-parameter health tracking that could be adapted for AGA therapy [176]. The development of self-powered sensing systems based on biofuel cells or triboelectric nanogenerators further enhances the potential for long-term, continuous monitoring without frequent battery changes or recharging [165,167]. These advances improve efficacy and safety, steering AGA care toward precision medicine [138,154,160].

In sum, real-time monitoring within intelligent MN systems could provide visibility into follicle status, drug exposure, therapeutic response, and adverse events during AGA treatment [138,154,160]. However, most AGA-oriented applications remain conceptual or at the prototype stage, and clinical validation is required before these systems can be incorporated into routine AGA management [138,141,154,160].

### 6.3 Future directions for intelligent microneedle systems as closed-loop platforms in hair regeneration

MN systems, as closed-loop platforms, hold substantial potential to reshape AGA therapy [138,154,160]. With advances in materials science, microelectronics, AI, and biotechnology, these systems will evolve toward greater precision, intelligence, safety, and convenience [138,154]. Recent reviews on intelligent soft robotics further highlight that microneedle arrays are evolving from passive delivery interfaces into biointegrated wearable systems that combine sensing, actuation, energy harvesting, and AI-assisted feedback control [177]. Beyond improving AGA outcomes, they may extend to other alopecia types, benefiting broader patient populations [154,160]. Future development will center on technological innovation, functional expansion, clinical translation, and industrialization [138,154,160]. The integration of nanosensors and nanotherapeutics into MN platforms is expected to drive the next generation of closed-loop systems for hair regeneration [155] (as conceptualized in Fig. 8A).

Technological innovation is the core driver [138,154]. In materials science, new biocompatible and degradable materials will enhance safety and comfort [138,141]. For example, MNs made from silk fibroin or hyaluronic acid can naturally degrade after delivery, eliminating the need for removal and improving user convenience [138]. The integration of advanced nanomaterials, such as metal-organic frameworks, quantum dots, and 2D materials like MXene and graphene, will endow MN platforms with enhanced sensing capabilities, including higher sensitivity, multiplexed detection, and novel signal transduction mechanisms [178,179]. In microelectronics, smaller, more sensitive, and more stable sensors will broaden biomarker coverage and yield richer data on the scalp microenvironment [138,154]. Flexible electronics will better conform to curved scalp surfaces, improving accuracy and comfort [154]. The development of stretchable and kirigami-inspired backing layers will enable MN patches to accommodate scalp movement and curvature while maintaining robust tissue adhesion [164,180]. In power systems, miniaturized, long-life, efficient energy solutions will address powering needs and prolong operation [138]. Innovations such as biofuel cells and energy harvesting may even enable self-powered systems without external sources [138,154]. Triboelectric nanogenerators that convert mechanical energy from hair movement or scalp motion into electrical power represent a promising approach for self-powered sensing and actuation in hair regeneration applications [164,165]. Together, these advances will deliver more efficient and reliable platforms [138,141,154].

Functional expansion is another key direction [154,160]. Future systems will move beyond drug delivery and monitoring to integrate multimodal therapies [138,154]. Phototherapy modules can be incorporated to stimulate follicles at specific wavelengths, thereby complementing pharmacologic delivery through physical stimulation [160,181]. Low-level laser therapy (LLLT), already validated in AGA, can be combined with MN platforms to achieve additive benefits [181,182]. Electrical stimulation can be integrated to promote hair growth via microcurrents [154]. Gene and cell therapies represent further frontiers: MN systems can precisely deliver gene-editing tools or stem cells to target sites for follicle repair and regeneration [123,128]. However, these modalities introduce additional safety and regulatory concerns, including off-target genetic effects, immune responses, long-term cell fate, tumorigenic risk, and manufacturing standardization. The integration of multifunctional nanomaterials enables the creation of "all-in-one" MN platforms capable of simultaneous therapy and monitoring. For instance, photothermal nanoparticles can be incorporated for near-infrared-triggered drug release while also serving as contrast agents for imaging-based monitoring [158,183] (Fig. 8C illustrates the principle of NIR-triggered drug release from microneedles). Similarly, theranostic MN patches combining therapeutic agents with sensing elements can provide real-time feedback on treatment efficacy, enabling truly closed-loop operation [155,184]. In parallel, enhanced onboard AI-based analytics will predict responses and optimize regimens using continuous monitoring data [138,154]. These expansions will transform intelligent MNs into multifunctional, integrated hair-regeneration platforms [138,154,160].

Clinical translation is pivotal [138,154]. Future work will emphasize rigorous validation and optimization under real-world conditions to establish effectiveness and safety [138]. Large, multicenter randomized controlled trials will benchmark intelligent MNs against standard therapies and provide high-level evidence [138,154]. Optimization of individualized protocols—derived from patient-specific response analytics—will refine parameter-adjustment strategies [154]. Long-term safety and effectiveness, including chronic biocompatibility and stability, warrant thorough assessment [138]. The translation of nanosensor-integrated MN platforms requires a comprehensive evaluation of their performance in human subjects, including sensor accuracy, temporal stability, and the correlation between measured biomarkers and clinical outcomes [19]. Regulatory pathways for combination products integrating drugs, devices, and biologics will need to be navigated carefully [19]. Applications across alopecia etiologies (e.g., alopecia areata, telogen effluvium) will be explored to expand indications [154,160]. These clinical efforts will lay the groundwork for broad adoption [138,154].

Industrialization is a necessary enabler [138,141]. Priorities include cost reduction and manufacturing efficiency to improve accessibility [138]; establishing standardized production and quality-control systems to ensure consistency and reliability [141]; and fostering clinical partnerships to accelerate deployment, collect real-world evidence, and inform iterative optimization [138]. Patient education and training will improve correct use and adherence [138,154]. Regulatory authorization and reimbursement are critical for scale-up; proactive engagement with regulators can expedite approval, and coverage policies will broaden access [138]. Scalable manufacturing processes for nanomaterial-integrated MN platforms, such as 3D printing and automated micromolding, will be essential for cost-effective production [185,186]. The development of quality control standards for the incorporation of nanomaterials and for sensor performance will be crucial for regulatory approval and market acceptance [19]. These measures will shepherd intelligent MN systems from lab to clinic [138,141].

Future development as closed-loop systems will still face challenges [138,154]: technical (miniaturization, integration, stability, reliability, sensor calibration, signal drift [166]), clinical (interindividual variability, long-term safety, effectiveness, and validation of biomarker surrogates [19]), industrial (cost control, scalable manufacturing, regulatory approval, standardization of nanomaterial-based components [19]), and ethical (data privacy, algorithmic fairness, equitable access). Addressing these issues will require multidisciplinary collaboration across academia, industry, and healthcare [138,154].

Nevertheless, prospects remain strong [138,154,160]. As technologies mature and clinical evidence accumulates, intelligent MN systems may become adjunctive or personalized options for AGA therapy, offering more precise and potentially safer treatment strategies [138,154]. Integration with emerging modalities—such as gene editing and stem-cell therapies—may ultimately enable true follicle regeneration and durable restoration, addressing alopecia at its root [123,128,138]. The convergence of nanotechnology, microelectronics, and artificial intelligence within closed-loop MN platforms promises to revolutionize not only hair regeneration but also the broader field of precision dermatology and regenerative medicine [155]. Such progress will not only transform AGA care but also propel the entire hair-regeneration field toward precision and personalized medicine [138,154,160].

## 7 Current limitations, translational challenges, and future research directions of microneedle-mediated hair regeneration

### 7.1 AGA-specific technical limitations and engineering bottlenecks

Balancing mechanical strength and penetration capability while avoiding intradermal microneedle (MN) fracture remains a key issue in MN design [7,24,117]. For androgenetic alopecia (AGA) therapy, this challenge is further amplified by the scalp-specific application environment. Residual hair shafts, sebum, scalp curvature, regional differences in skin thickness, and patient-dependent application force may all reduce patch–skin contact, resulting in uneven insertion depth and compromised dose reproducibility [14,83]. Therefore, MN performance should not be evaluated solely by fracture force or insertion depth in flat skin models, but should also be assessed using hair-bearing scalp models, flexible backing layers, adhesion tests, and applicator-assisted insertion strategies [7,14,24,83,177]. Shin et al. developed PLGA microsphere-loaded candle microneedles (CMNs) to address insertion and adhesion challenges on hair-bearing skin [14,24],. This example highlights an important translational issue: scalp-adapted MNs require co-optimization of needle geometry, patch adhesion, applicator design, and user-independent insertion performance, rather than relying only on tip sharpness [14,83].

Payload stability, especially for biologics such as growth factors, enzymes, nucleic acids, and cell-derived products, remains another major challenge; platelet-rich plasma (PRP) MNs have employed temperature-induced gelation to preserve bioactivity [106]. However, compared with small-molecule-loaded MNs, biologic-loaded MNs face more stringent stability challenges, because their biological activity may decline during micromolding, drying, crosslinking, lyophilization, terminal sterilization, storage, and transportation [187,188]. For PRP, extracellular vesicles, conditioned medium, stem cell-derived products, and nucleic acid payloads, future studies should not only report loading efficiency and release profiles. Still, they should also characterize post-fabrication potency, batch-to-batch variability, residual bioactivity after storage, and degradation pathways [106,112,[187], [188], [189], [190]]. Without these assays, apparent hair-regenerative efficacy in animal models cannot be reliably translated into the reproducible manufacture of biological products [[187], [188], [189], [190]].

Immune rejection and immune activation are particularly important but under-discussed barriers for biologic- and cell-derived MN systems. Even when MNs are applied locally, repeated exposure to allogeneic extracellular vesicles, conditioned medium, stem cell-derived factors, recombinant proteins, viral or nonviral gene-editing payloads, or residual cellular components may induce local inflammation, anti-product antibodies, complement activation, delayed-type hypersensitivity, or alteration of the perifollicular immune-privileged state [112,[187], [188], [189], [190], [191]]. This issue is especially important for alopecia areata, in which follicular immune privilege is already compromised. Still, it is also relevant to chronic AGA because patients may require repeated treatment for months or even years [112,191]. Therefore, future studies of biologic-loaded MNs should incorporate immunogenicity testing, cytokine profiling, analysis of local lymphocyte/macrophage infiltration, complement activation markers, detection of anti-vector or anti-protein antibodies, and scalp histopathology after repeated dosing, rather than relying solely on short-term erythema or gross toxicity observations [187,189,190].

Process control and consistency affect product quality and therapeutic efficacy; machine learning-driven formulation design identified optimized materials with high hardness and rapid dissolution after only 18 experiments [153,192]. However, formulation optimization should be further linked to clinically relevant outputs, including follicular deposition, local pharmacokinetics, scalp tolerability, insertion reliability after repeated use, and storage stability [30,153]. Other obstacles include limited drug loading, control over release kinetics, and scalable manufacturing with quality assurance [5,29,193]. For chronic AGA, insufficient drug loading may necessitate frequent administration, whereas excessive or poorly controlled release may increase irritation or systemic exposure. Therefore, release kinetics should be optimized to avoid burst release, premature leakage, incomplete release, and prolonged exposure beyond the target follicular response window [5,29,30,193].

Scalability is not a generic manufacturing issue, but a concrete translational bottleneck. Many laboratory MN systems rely on low-throughput micromolding, vacuum filling, centrifugation, manual casting, long drying times, fragile nanocarrier integration, or low-temperature processing suitable for biologics [14,83,193]. These procedures are difficult to translate into current good manufacturing practice (cGMP) production because they may cause batch-to-batch variation in needle height, tip sharpness, drug distribution, residual moisture, mechanical strength, dissolution rate, and biological potency [14,83,193]. Sterilization is another major pain point: heat sterilization, ethylene oxide, gamma irradiation, and electron-beam sterilization may each cause polymer deformation, drug degradation, damage to extracellular vesicles or proteins, or altered nanomaterial properties [187,189,193]. Accordingly, scalable MN development should establish process analytical controls, in-line inspection of geometry and dose uniformity, validated sterilization or aseptic manufacturing strategies, and packaging systems that control humidity while maintaining mechanical strength [14,83,193].

Advanced MN systems integrating nanomaterials, biologics, sensors, or multiple drugs further increase the complexity of quality control. Critical quality attributes should include needle geometry, insertion efficiency, fracture resistance, dose uniformity, residual solvents or monomers, microbial burden, endotoxin level, sterilization compatibility, release kinetics, and retained biological activity after manufacturing and storage [83,187]. Recent disease-tailored MN frameworks suggest that MN design should shift from “technology-generalized” platforms to disease-specific engineering based on the disease microenvironment, drug properties, and therapeutic objectives [18,194]. For AGA, a successful MN design should not be judged solely by skin penetration. Still, it should also be evaluated for reproducible follicular targeting, immunocompatibility, scalable manufacturability, and long-term repeated-use performance [14,83,193].

### 7.2 Translational and clinical application barriers

A major translational challenge lies in the mismatch between the complexity of preclinical MN systems and the simplicity of current clinical practice for alopecia treatment. Most advanced hair-regenerative MN platforms, including dissolvable MNs, stimuli-responsive MNs, nanocarrier-integrated MNs, biologic-loaded MNs, and closed-loop concepts, remain at the proof-of-concept or animal-study stage. In contrast, clinical research in alopecia still primarily focuses on mechanical MNs, radiofrequency MNs, or simple combinations with topical drugs. This bench-to-bedside gap indicates that technological novelty should not be equated with clinical maturity [[195], [196], [197]]. A recent review on MNs for alopecia also noted that basic research primarily focuses on dissolvable MN patches, stimuli-responsive systems, and complex nanocarriers. In contrast, clinical trials are largely focused on mechanical rollers, radiofrequency MNs, and topical drug combinations [196197198].

Safety evaluation must assess biocompatibility, degradation products, and local/systemic toxicity under long-term use [103]. Hu et al. showed that Pt MNs formed transient and reversible skin channels without Pt residues in major organs, suggesting favorable safety under the reported experimental conditions [103]. However, chronic AGA treatment requires a more comprehensive safety assessment than short-term animal observation. Repeated puncture may alter scalp barrier recovery, increase susceptibility to irritation or infection, and modify perifollicular inflammatory status [83,197]. Anti-androgen MNs require assessment of systemic hormonal leakage and reproductive safety, whereas MNs containing nanozymes or metallic components require long-term studies on clearance and accumulation [103]. For biologic-, extracellular vesicle-, and gene-editing-loaded MNs, immune rejection and immune activation should be considered major translational risks rather than minor safety details [112,187,189].

Immune-related risks should be evaluated in a product-specific manner. Autologous PRP may carry a relatively low risk of rejection, but still exhibits interpatient variability and activation-state-dependent differences. Allogeneic extracellular vesicles or stem cell-derived products may be more suitable for scalable production, but introduce donor-derived variability, residual host cell proteins, nucleic acids, major histocompatibility complex-related signals, and potentially immunogenic contaminants [112,187,189,191]. Recombinant growth factors or enzymes may induce anti-product antibodies after repeated exposure, whereas viral or nonviral nucleic acid delivery systems may activate innate immune sensors [[187], [188], [189], [190]]. Therefore, immune safety evaluation should include source control, donor screening, identity and purity testing, residual host-cell component analysis, cytokine release evaluation, complement activation, anti-product antibody monitoring, and local histological assessment after repeated dosing in scalp-relevant models [[187], [188], [189], [190]].

Efficacy validation requires well-designed clinical trials to characterize human pharmacokinetics, biodistribution, and therapeutic outcomes [30,191]. Pharmacokinetic differences between MN-mediated protein delivery and subcutaneous injection must also be considered [191]. Importantly, many preclinical studies use hair coverage, pigmentation, or anagen induction in mouse models as endpoints. Still, these outcomes do not directly correspond to human terminal hair density, hair shaft diameter, global photographic assessment, patient-reported outcomes, or durability after treatment discontinuation [30,191]. For multicomponent systems, single-agent control groups and quantitative interaction analyses should be incorporated to determine whether improved outcomes are additive, complementary, or truly synergistic [30,191].

Patient adherence, usability, and cost-effectiveness will determine real-world application [25,191]. Clinical observations suggest that serum-assisted MN combination therapy can reduce treatment frequency and improve efficacy [8]. However, clinical applicability depends on whether patients can use MN systems safely and consistently under real scalp conditions. Hair interference, visible residue, pain, bleeding, application time, training requirements, patch retention, and compatibility with daily hair care may determine adherence as much as biological efficacy [14,83]. Therefore, future studies should include usability testing, patient-reported outcomes, and real-world adherence metrics, rather than focusing solely on hair-count endpoints [83].

Regulatory approval, intellectual property protection, and scalable manufacturing are crucial [25,35,193]. From a regulatory perspective, MN systems that combine devices with drugs, biologics, nanomaterials, sensors, or energy-triggered actuation modules are likely to be classified as complex combination products. Their development requires a clear definition of the primary mode of action, critical quality attributes, release specifications, sterilization strategy, packaging stability, and human factors performance [60,83,198]. Translational microneedle array patch (MAP) literature emphasizes that microbial attributes, mechanical properties, drug stability, and dose uniformity are key quality attributes in regulatory development [83,198]. Furthermore, as of early 2026, no MN-based combination drug product has received marketing approval from the US FDA or EMA, underscoring the regulatory gap between technical feasibility and approved clinical products [83,191,198].

Scalability should also be evaluated from economic and operational perspectives. Even if a system is effective in small-animal studies, it may still fail commercially if it depends on expensive nanomaterials, low-yield biologics, multistep manual assembly, cold-chain logistics, individualized preparation, or specialized clinical equipment [14,83,193]. For AGA, a disease that may require long-term repeated treatment over relatively large target areas, unit patch cost, manufacturing yield, shelf life, applicator compatibility, and reimbursement feasibility may become decisive factors [193,199,200]. Therefore, future translational studies should report not only efficacy, but also manufacturing yield, batch failure rate, storage stability, post-sterilization recovery, estimated unit cost, and compatibility with automated production [83,193,199,200].

### 7.3 Future technological innovations required for bench-to-bedside translation

Future development should shift from technology-driven MN design toward AGA-adapted engineering. Next-generation MNs should not simply increase structural complexity. Still, they should be designed around scalp anatomy, hair density, follicular target depth, disease stage, payload properties, dosing frequency, and intended treatment duration [83,193,[201], [202], [203]]. Disease-tailored MN frameworks emphasize that microenvironmental differences, drug properties, and therapeutic goals should jointly determine device architecture [18,194]. For AGA, this means developing scalp-adapted backing layers, applicators capable of separating hair shafts or overcoming hair interference, needle lengths suitable for perifollicular deposition, moisture-protective packaging, and formulations suitable for repeated use in outpatient or home settings [14.79,202-204].

Future priorities include highly specific targeting, such as AR-PROTAC MNs for androgen receptor degradation [1,18,203]. However, highly specific interventions should incorporate safety boundaries during development. AR degradation, gene regulation, or immune modulation may provide strong local efficacy but require assessment of local selectivity, reversibility, off-target tissue effects, effects on nonfollicular androgen-responsive cells, and long-term recurrence across multiple hair cycles [18,112,187,190,203].

New materials and fabrication technologies, including 3D printing and two-photon polymerization, may enhance functionality and production efficiency [25,83,118]. However, future material innovation should place manufacturability on equal footing with biological performance. High-resolution technologies should be evaluated not only by geometric precision but also by printing speed, resin or polymer biocompatibility, residual monomers, sterilization tolerance, batch reproducibility, unit cost, and compatibility with automated packaging [83,193,198]. For large-scale production, modular designs and platform materials may be more translationally feasible than highly individualized architectures that are difficult to validate and scale up [83,193,198].

AI-assisted design and optimization of materials, structures, and individualized treatment regimens represent another important direction [105,149,153,187,204]. Previous studies have shown that machine learning can accelerate the optimization of material composition [153]. However, AI should not optimize only hardness or dissolution time; future models should integrate needle mechanics, scalp contact behavior, local pharmacokinetics, follicular deposition, release kinetics, immune response signals, and clinical endpoints [52,153,193,205]. AI-optimized MNs will require standardized datasets, transparent algorithms, external validation, and predefined safety constraints before model recommendations can guide clinical product development [83,205].

Integrated theranostic MNs that combine biosensing and precision delivery may support personalized, long-acting treatment strategies [25]. MN–wearable system integration could enable real-time monitoring and dynamic dosing. At the same time, nanosensors and flexible electronics may provide feedback on inflammatory mediators, drug exposure, scalp hydration, or local microenvironmental status [25,83,206]. However, closed-loop systems should be validated in stages: first, by confirming sensing accuracy and stability in scalp-relevant interstitial fluid (ISF); second, by demonstrating that measured biomarkers correlate with clinical hair growth outcomes; and finally, by testing whether automatic dose adjustment truly improves efficacy or safety compared with fixed regimens [83,206]. Personalized strategies, long-acting systems, and noninvasive activation will become core directions [1,7,24,52]. Xu et al. emphasized the roles of MNs, liposomes, and ionic liquids in transdermal AGA therapy [1].

For biologic and cell-derived therapies, future innovation should focus on immune-compatible and scalable product design. Autologous approaches may reduce immune rejection, but are costly, variable, and difficult to standardize; allogeneic or off-the-shelf products may improve scalability but require stronger immune safety control [112,[187], [188], [189], [190], [191]]. Therefore, future MN–biologic platforms should establish donor qualification, standardized isolation and purification, potency assays, release criteria, immunoreactivity testing, and storage conditions that preserve function without excessive reliance on cold-chain logistics [[187], [188], [189], [190]].

### 7.4 Clinical translation roadmap and future research priorities

A practical bench-to-bedside translational roadmap should involve continuous validation across material, device, biological, manufacturing, regulatory, and clinical levels. At the material level, future studies should report standardized mechanical strength, insertion efficiency, dissolution or swelling kinetics, drug stability, residual moisture, sterility, and storage stability [58,83]. At the device level, scalp-specific testing models should be established to evaluate insertion through hair-bearing skin, patch adhesion under sebum and curvature conditions, dose uniformity, applicator compatibility, and user-independent application performance [14,83,[201], [202], [203]]. At the biological level, repeated-dose safety, local immunogenicity, nanomaterial clearance, immune-rejection risk, and biomarker validity should be assessed in models that more closely reflect chronic AGA treatment [103,112,190,191].

At the manufacturing level, future MN studies should move beyond manually fabricated prototypes and report scale-up-related parameters, including batch size, production yield, drying time, post-sterilization recovery, packaging requirements, shelf life, and cost drivers [18,83,193]. At the regulatory level, developers should define critical quality attributes early, including microbial burden, endotoxin level, geometry, mechanical strength, dose uniformity, drug or biologic potency, release kinetics, and human factors performance [83,187,198]. These data form the basis for translating MN systems from academic prototypes into regulated products [75,83,193,207].

At the clinical level, early trials should prioritize relatively simple and clinically feasible systems, such as MN-assisted delivery of minoxidil or finasteride, before gradually advancing to complex, multicomponent, nanomaterial-integrated, biologic-loaded, or closed-loop platforms [83,195,197,198]. Outcome measures should include not only hair density and hair shaft diameter but also treatment adherence, scalp tolerability, pain, cosmetic acceptability, patient-reported satisfaction, relapse after discontinuation, and comparative effectiveness relative to standard topical or systemic therapies [30,52,197]. For multicomponent systems, appropriate single-agent groups should be included whenever feasible to determine whether the benefit of combination therapy is additive, complementary, or truly synergistic [30,52,197].

Combining MNs with electrospun nanofibers can create synergistic platforms for wound healing and tissue regeneration; nanofibers can mimic the extracellular matrix (ECM) and serve as drug reservoirs, whereas MNs enable precise deposition [83]. For alopecia applications, such hybrid systems should be evaluated not only for regenerative potential but also for follicular retention, biodegradability, scalp comfort, sterilization compatibility, and manufacturing reproducibility [75,83,207]. MN–wearable system integration supports closed-loop architectures for real-time monitoring and dynamic dosing, advancing personalized medicine [25,206]. However, wearable integration must address power supply, sensor drift, data security, long-term adhesion, and patient usability before it can support routine AGA management [83,206]. MN–nanomedicine integration can enable multifunctional local delivery; Chen et al. developed a dual-targeted nanotherapy that alleviated oxidative stress and activated autophagy to promote regeneration [69,147]. 3D printing supports complex, multilayered, and customizable MN arrays for personalized treatment [149,193]. However, increased structural complexity must be balanced against dose uniformity, sterilization, storage stability, regulatory testing burden, and cost [75,83,193,199].

MNs combined with nanorobots may enable ultraprecise local drug delivery and tissue repair [[96], [97], [98],^192^]. At present, such nanorobot or microrobot strategies in alopecia remain largely conceptual and should be positioned as long-term technological inspiration rather than near-term clinical solutions [193,208,209]. Integration with stem cell and gene-editing technologies may further expand MN capabilities [112,191,207]. Long et al. reviewed MNs for in situ tissue regeneration across multiple indications, including skin, cornea, and myocardium [191]. However, these therapies require particularly rigorous assessment of immune rejection, off-target effects, insertion-site retention, long-term cell fate, ectopic tissue reactions, and tumorigenic risk [112,[187], [188], [189], [190], [191]].

Overall, MN-mediated hair regeneration is evolving from simple barrier disruption toward disease-adapted, multifunctional, and potentially closed-loop therapeutic systems. The most important future task is not merely to create more complex devices, but to establish clinically translatable platforms that are reproducibly manufacturable, mechanistically validated, immune-compatible, standardized in quality attributes, cost-effective, and capable of demonstrating patient benefit. Only through such a staged pathway can MN-based hair regeneration technologies progress from promising laboratory systems to practical, scalable, and safe therapeutic options for alopecia [14,83,193,[201], [202], [203]].

## 8 Conclusions

This review summarizes recent advances in MN-mediated intelligent combination delivery systems for hair regeneration, with a particular focus on how MN platforms integrate material design, follicular delivery, therapeutic synergy, stimuli responsiveness, and closed-loop regulation. Compared with conventional topical or systemic therapies, MNs provide a unique therapeutic interface for alopecia treatment: they can not only bypass the stratum corneum barrier, enhance local follicular exposure, and reduce unnecessary systemic distribution, but also activate wound-repair and regeneration-related signaling through mechanical stimulation. As highlighted in this review, MN-based strategies have evolved from simple penetration-enhancing tools into multifunctional platforms capable of delivering small-molecule drugs, natural products, biologics, extracellular vesicles, nanozymes, growth factors, collagen-related components, and physical stimuli to the follicular microenvironment. These advances are particularly important because alopecia, especially androgenetic alopecia (AGA), is not governed by a single pathological pathway, but rather results from the combined effects of androgen signaling, oxidative stress, inflammation, impaired angiogenesis, extracellular matrix remodeling, and insufficient activation of hair follicle stem cells.

The central perspective of this review is that the value of MNs in hair regeneration should not be understood merely as enhanced transdermal delivery, but rather as a strategy for reconstructing alopecia therapy around the follicular microenvironment. In this context, the significance of intelligent combination delivery lies not simply in loading multiple components into a single patch, but in selecting therapeutic components based on their complementary biological functions. Antioxidant therapy combined with pro-angiogenic support, growth factor supplementation combined with collagen or extracellular matrix remodeling, anti-androgen regulation combined with microenvironmental repair, and immunomodulation combined with regenerative stimulation all represent more biologically rational directions for synergistic design. Therefore, future MN-based combination therapies should not be evaluated solely by visible hair regrowth or hair coverage, but should also be assessed by whether they achieve coordinated, durable, and mechanistically supported restoration of the follicular microenvironment.

Intelligent stimuli-responsive MNs and closed-loop MN systems further extend this therapeutic logic, shifting treatment paradigms from fixed dosing toward adaptive intervention. ROS-, enzyme-, pH-, temperature-, light-, ultrasound-, magnetic-, and electrically responsive MNs offer opportunities to generate release profiles that more closely match local pathological signals. Meanwhile, MN platforms with sensing functions and AI-assisted analytical capabilities suggest a future direction in which individualized dosing could be guided by scalp biomarkers, drug exposure, inflammatory status, and follicular activity. However, these technologies should be interpreted in light of their different levels of maturity: delivery enhancement and rational combination therapy are closer to practical translation, whereas fully autonomous closed-loop systems remain an emerging direction in alopecia and are still largely in the preclinical stage. Making this distinction helps avoid overstatement of the field while preserving its long-term innovative potential.

Looking ahead, the key task is to translate MN-mediated hair regeneration from successful experimental demonstrations into clinically useable, reproducible, and scalable therapeutic systems. This does not simply mean adding more functions to a patch. Future studies must address scalp-specific insertion under hair-bearing conditions, follicular targeting accuracy, payload stability during fabrication and storage, immune compatibility of biologic or cell-derived payloads, release reproducibility, standardized efficacy endpoints, patient usability, scalable manufacturing, sterilization compatibility, and regulatory feasibility. In particular, increasing device complexity must be supported by clear therapeutic benefit, stable quality control, and manufacturability; otherwise, simpler MN-assisted therapeutic strategies may be more clinically realistic.

Overall, MN-mediated intelligent combination delivery represents an enabling platform that bridges the multifactorial biology of alopecia with precise local intervention. Its near-term clinical value may lie in improving existing therapies by enhancing follicular delivery, reducing treatment burden, and enabling rational synergistic combinations. Its long-term potential lies in developing intelligent systems capable of sensing, interpreting, and regulating the follicular microenvironment in a patient-specific manner. If future research can balance mechanistic complexity with safety, usability, scalability, and rigorous clinical validation, MN platforms may become an important adjunctive or personalized therapeutic option for hair regeneration, rather than remaining only a promising laboratory technology.

## CRediT authorship contribution statement

**Liang Lan:** Writing – original draft, Visualization, Conceptualization. **Yiyu Wang:** Writing – original draft, Visualization, Conceptualization. **Bin Xue:** Project administration. **Peng Chen:** Project administration.

## Ethics approval

This article does not contain any studies with human participants or animals performed by any of the authors.

## Funding

This work was funded by the 10.13039/501100001809Natural Science Foundation of China (Grant Nos. 82202469), 10.13039/501100005230Natural Science Foundation of Chongqing, China (Grant No. CSTB2023NSCQ-MSX0412), and 10.13039/501100007957Chongqing Municipal Education Commission Youth Project(Grant No. KJ2024004109408467)

Declarations.

## Declaration of competing interest

The authors declare that they have no known competing financial interests or personal relationships that could have appeared to influence the work reported in this paper.

## Data Availability

No data was used for the research described in the article.
